# Liquid biopsy biomarkers for early detection of gastrointestinal cancers: Current landscape and emerging technologies

**DOI:** 10.1002/ctm2.70594

**Published:** 2026-03-22

**Authors:** Muhammad Anees, Christopher Sherry, Hyun Y. Park, Erin E. Grayhack, Arul Goel, Alisha F. Khan, Ashten Omstead, David L. Bartlett, Ajay Goel, Neda Dadgar, Patrick L. Wagner, Ali H. Zaidi

**Affiliations:** ^1^ Allegheny Health Network Cancer Institute Allegheny Health Network Pittsburgh Pennsylvania USA; ^2^ Department of Molecular, Cellular & Developmental Biology University of California Santa Barbara Santa Barbara California USA; ^3^ Department of Molecular Diagnostics and Experimental Therapeutics Beckman Research Institute of City of Hope, Biomedical Research Center Monrovia California USA

**Keywords:** cell‐free DNA methylation, circulating tumour cells (CTCs), circulating tumour DNA (ctDNA), early cancer detection, extracellular vesicles/exosomes, fragmentomics, gastrointestinal cancers, liquid biopsy, multi‐cancer early detection (MCED), tumour‐educated platelets (TEPs)

## Abstract

Early detection of gastrointestinal (GI) cancers remains a critical unmet clinical need, as most patients are diagnosed at advanced stages when prognosis is poor. Liquid biopsy has emerged as a transformative approach for minimally invasive cancer detection by analysing tumour‐derived analytes in blood and other body fluids. Recent advances in circulating tumour DNA (ctDNA) sequencing, cell‐free DNA methylation profiling, fragmentomics, extracellular vesicle and exosome characterisation, circulating tumour cell isolation and tumour‐educated platelets have markedly improved sensitivity and specificity for detecting incipient malignancies. Despite these advances, sensitivity in stage I disease remains limited due to low tumour burden and minimal analyte scaling, resulting in false‐negative results for small or indolent lesions. In addition, clonal haematopoiesis derived alterations can confound mutation‐based assays, highlighting the need for epigenetic and multi‐analyte approaches to improve specificity. Ultimately, widespread clinical adoption will require standardised, prospective trials demonstrating diagnostic accuracy and a reduction in cancer‐specific mortality. Multi‐analyte and machine learning‐driven approaches, integrating DNA, RNA, protein and epigenomic signals, are now in late‐stage clinical trials and poised for clinical translation. United States Food and Drug Administration approvals of blood‐based colorectal cancer screening tests and laboratory‐developed assays for hepatocellular carcinoma exemplify the translational momentum in this field. Here, we review the current landscape of liquid biopsy biomarkers for GI cancers, emphasising technological innovations, clinical performance and ongoing trials. We also discuss key challenges, including sensitivity in stage I disease, specificity amidst clonal haematopoiesis and integration with established screening paradigms. The continued evolution of assay technologies and translational research heralds a paradigm shift towards precision early detection of GI cancers, with the potential to substantially reduce mortality through earlier intervention.

**Key points:**

- Liquid biopsy technologies are advancing rapidly for early detecion of GI cancers, using ctDNA, methylation profiling, fragmentomics, EVs, CTCs, and TEPs.
- Limited sensitivity in stage I disease remains a key barrier, largely due to low tumor burden and analyte scarcity.
- Clonal hematopoieses confounds mutation‐based assays, emphasizing the need for epigenetic and multi‐analyte strategies to improve specificity.
- Multi‐analyte, machine‐learning‐driven platforms are nearing clinical translation, supported by late‐stage trials and recent FDA approvals.

## Graphical abstract

This narrative review provides a comprehensive overview of liquid biopsy biomarkers for early detection of gastrointestinal cancers focusing on technological innovations, clinical performance and ongoing clinical trials.

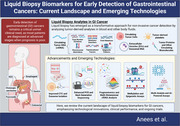

## 1 INTRODUCTION

Gastrointestinal (GI) cancers – including malignancies of the colon, rectum, stomach, oesophagus, pancreas, liver and biliary tract are among the leading causes of cancer mortality worldwide.^1^ A major challenge is that many GI tumours are diagnosed at advanced stages due to lack of obvious early symptoms or effective screening tools. Traditional screening (such as endoscopy for colorectal or gastric cancer) can reduce mortality but is invasive, costly and limited to certain populations (age appropriate determined by the United States Preventive Service Task Force, patients with high risk factors according to their specific disease state consortium guidelines, or with symptomatology in which benefits to screening outweigh the risk). Liquid biopsy has emerged as a promising, minimally invasive approach to detect cancers earlier by analysing tumour‐derived biomarkers in body fluids.^2^, ^3^ Liquid biopsies evaluate circulating tumour components like DNA fragments, whole cells or vesicles that tumours shed into systemic circulation.^4^, ^5^ Compared with tissue biopsy, liquid biopsy is safer, repeatable and may better capture tumour heterogeneity.

Notably, despite substantial technological maturation, only a small number of blood‐based assays, most prominently methylated SEPT9 and the recently approved Shield test for colorectal cancer, have achieved regulatory approval for screening indications.^6^, ^7^ This limited adoption reflects both technical challenges, regulatory inertia and the high evidentiary threshold required to demonstrate benefit in asymptomatic populations. In contrast to diagnostic or treatment monitoring applications, population‐based screening requires large prospective studies with long‐term follow up to establish mortality reduction, cost effectiveness and acceptable false positive rates. These implementation barriers have delayed integration of liquid biopsy assays into public health screening programs, despite their growing clinical promise.

**FIGURE 1:**
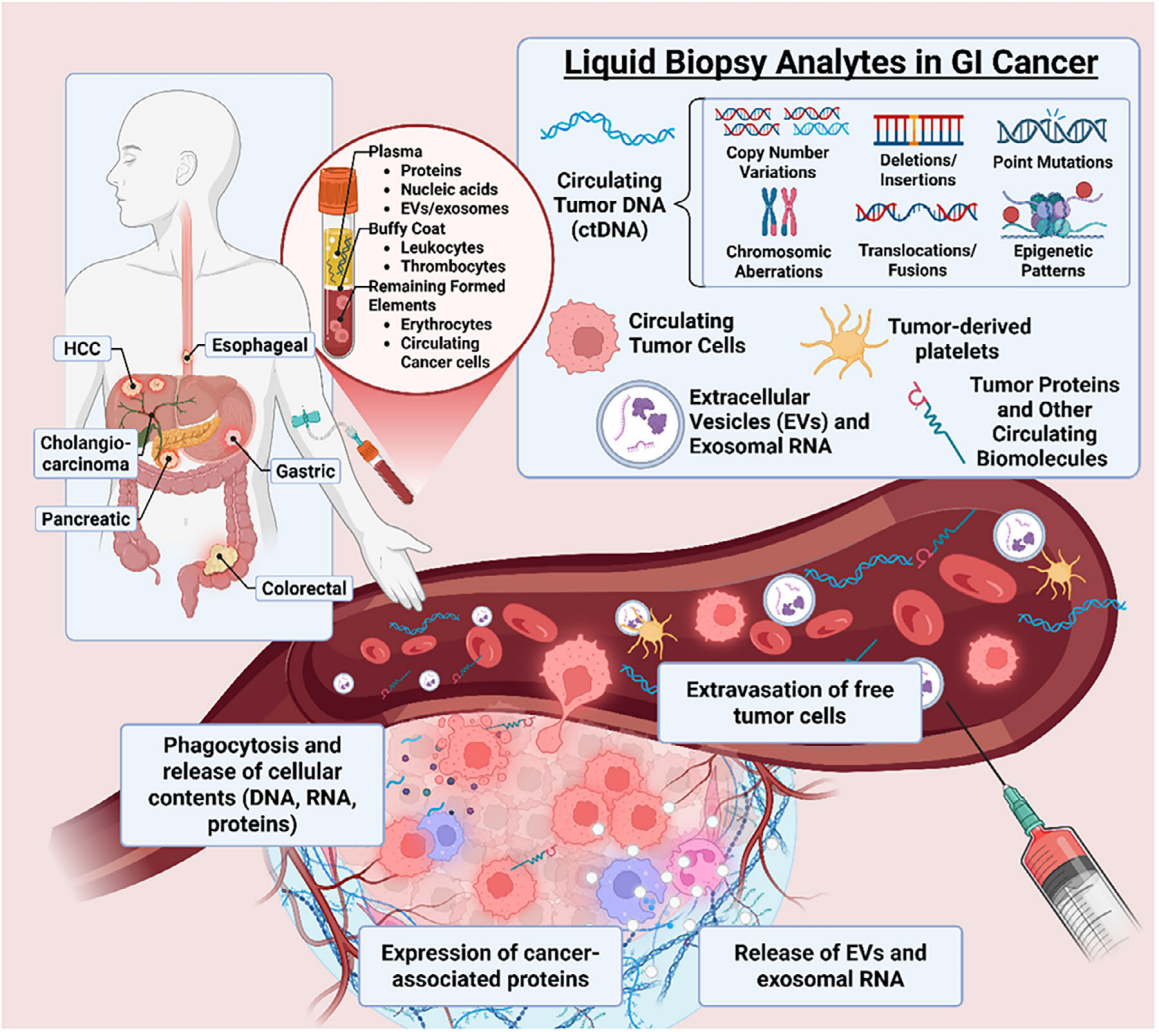
Liquid biopsy analytes and their origin in gastrointestinal cancers. This figure depicts the various gastrointestinal (GI) cancers (HCC, oesophageal, gastric, pancreatic, cholangiocarcinoma, colorectal) and the different tumour‐derived analytes found in blood that are utilised in liquid biopsy. These include circulating tumour DNA (ctDNA) carrying genetic and epigenetic alterations, circulating tumour cells (CTCs) that extravasate from the primary tumour, extracellular vesicles (EVs) and exosomal RNA (such as microRNAs), tumour‐derived platelets (TEPs), and other tumour proteins and circulating biomolecules. The diagram also illustrates the processes of phagocytosis, release of cellular contents and expression of cancer‐associated proteins contributing to these circulating analytes. *Abbreviations*: HCC, hepatocellular carcinoma; GI, gastrointestinal; ctDNA, circulating tumour DNA; EVs, extracellular vesicles; RNA, ribonucleic acid; CTCs, circulating tumour cells; DNA, deoxyribonucleic acid; miRNA, microRNA; TEPs, tumour‐educated platelets.

This review discusses the current landscape of liquid biopsy biomarkers for early detection of GI cancers, highlighting emerging technologies that improve assay sensitivity (Figure 1) and outlining the translational progress including ongoing clinical trials.

## 2 LIQUID BIOPSY ANALYTES IN GI CANCERS

Liquid biopsy analytes encompass a diverse group of circulating tumour‐derived components that reflect complementary aspects of tumour biology, including genetic alterations, epigenetic regulation, cellular dissemination and tumour–host interactions. For clarity, these analytes are classified based on their biological origin, cell‐free nucleic acids, intact tumour cells, extracellular vesicles (EVs), tumour‐educated platelets (TEPs) and circulating proteins, each offering distinct advantages and limitations for early cancer detection. This framework facilitates comparison across biomarker classes and highlights opportunities for integrative, multi‐analyte approaches. Table 1 summarises the performance metrics, availability of validated tests and phase of clinical development for different liquid biopsy analytes across GI cancers.

**TABLE 1:** Comparative analysis of liquid biopsy analytes for gastrointestinal cancer detection.

Analyte	Performance metrics	Availability of validated assays	Phase of clinical development	References
ctDNA	Sensitivity: 40–70% in Stage I/II; improved to ∼90% using methylation markers. Specificity: >95%	US FDA‐approved for CRC screening (Shield, Epi proColon).	Clinical implementation (CRC only); late clinical validation for other GI cancers	^7^ ^,^ ^10^
CTCs	Sensitivity: 40–85% (variable); lower in early stages. Specificity: ∼99%	CellSearch US FDA‐cleared for prognostic use in metastatic CRC only; no validated screening assays	Early clinical validation	^11^ ^,^ ^13^
EV‐derived RNA	Sensitivity: 70–96% for early‐stage gastric and liver cancers. Specificity: 85–90%	EvoLiver (HCC surveillance) holds 2025 US FDA breakthrough status; DESTINEX (early gastric cancer)	Early → late clinical validation (cancer‐type dependent)	^14^, ^17^
TEPs	Sensitivity: >90% excellent for identifying cancer signal origin (CSO) but highly experimental for primary screening. Specificity: variable	No standalone commercial test; primarily research platforms (e.g., ThromboDx/Illumina)	Discovery/preclinical → early clinical validation	^18^
Protein biomarkers	Sensitivity: ∼30%‐60%. Misses many early‐stage cancers. Specificity: moderate	Widely available clinical immunoassays (CEA, CA19‐9, AFP) but not validated for early detection	Clinical implementation (non‐screening use); early validation for screening	^19^

Abbreviations: CRC, colorectal cancer; ctDNA, circulating tumour DNA; CTCs, circulating tumour cells; EV‐derived RNA, extracellular vesicle‐derived ribonucleic acid; US FDA, United States Food and Drug Administration; GI, gastrointestinal; CEA, carcinoembryonic antigen; AFP, alpha‐fetoprotein; HCC, hepatocellular carcinoma; TEPs, tumour‐educated platelets.

### 2.1 Circulating tumour DNA

Circulating tumour DNA (ctDNA) are the fragments of tumour‐derived DNA circulating freely in the blood (as part of the cell‐free DNA pool). These fragments carry tumour‐specific genetic or epigenetic alterations. Because ctDNA often represents a tiny fraction (<.1% in early stage) of total cell‐free DNA, ultrasensitive techniques are required. ctDNA can reveal mutations like AKT1, APC, BRAF, CDKN2A, CTNNB1, EGFR, FBXW7, FGFR2, GNAS, HRAS, KRAS, NRAS, PIK3CA, PPP2R1A, PTEN, TP53, TOP2A, RNF43 (NCT05991947) (Table S1), copy number changes and methylation patterns associated with GI cancers. Its short half‐life (around 2 h) means ctDNA reflects real‐time tumour status.^20^ Importantly, ctDNA is present in virtually all GI cancer types and is a central target for blood‐based early detection. As a minimally invasive technique, ctDNA monitoring allows for serial sampling and monitoring of tumour dynamics, which is particularly useful for early diagnosis, risk stratification and detection of minimal residual disease (MRD). ctDNA can reflect the molecular heterogeneity of tumours and may detect actionable mutations or resistance mechanisms when tissue biopsy is not feasible,^21^, ^22^ allowing for early initiation of appropriate and effective treatment paradigms (Table S1). However, ctDNA also has notable disadvantages for early detection. Sensitivity is limited in early‐stage disease due to low tumour burden and minimal DNA shedding, resulting in a high false‐negative rate for stage I cancers.^23^ Technical challenges include the need for highly sensitive assays to detect low‐frequency variants and pre‐analytical/analytical variability can affect reproducibility.^24^, ^25^, ^26^ False positives may arise from clonal haematopoiesis or non‐malignant sources of cell‐free DNA (cfDNA), complicating interpretation.^23^

#### 2.1.1 Mechanistic validation of ctDNA targets using genetic perturbation models

A central question in the clinical application of ctDNA for early cancer detection is whether recurrent genomic alterations detected in plasma truly reflect tumour‐specific biology rather than stochastic DNA release or non‐malignant processes. The key ctDNA‐detected genes are summarised in Table S1. Extensive genetic perturbation studies in cell lines and animal models provide strong mechanistic validation for many of the genes recurrently detected in ctDNA across GI cancers, confirming their causal role in tumour initiation, progression and DNA shedding. Loss‐of‐function studies of APC, the most frequently altered gene in colorectal cancer, have been particularly informative. Germline and intestine‐specific APC knockout models consistently demonstrate spontaneous adenoma formation through constitutive Wnt/β‐catenin activation, establishing APC loss as the initiating event in the adenoma–carcinoma sequence.^27^, ^28^ Importantly, tumour burden in these models correlates with increased ctDNA levels, supporting the concept that early neoplastic lesions driven by APC loss are biologically capable of releasing detectable tumour‐derived DNA into circulation. These findings directly support the use of APC‐associated mutations and downstream methylation signatures as ctDNA markers for early colorectal neoplasia. Similarly, TP53 loss has been functionally validated as a driver of genomic instability and tumour progression in multiple GI cancer models. Conditional Trp53 knockout in intestinal epithelium accelerates tumourigenesis when combined with APC loss, leading to increased chromosomal instability, higher proliferation rates and enhanced cell turnover.^29^, ^30^ These biological effects provide a mechanistic explanation for the increased abundance and diversity of TP53 mutations observed in ctDNA as tumours progress, and they underscore why TP53 alterations are frequently detected even in early‐stage GI cancers via highly sensitive assays. Oncogenic activation of the RAS–MAPK pathway has likewise been validated through genetic perturbation models. While complete knockout of KRAS or BRAF is embryonically lethal, tissue‐specific expression of mutant alleles (e.g., KRASG12D or BRAF V600E) in intestinal and pancreatic epithelium induces hyperproliferation, dysplasia and invasive carcinoma.^31^, ^32^ These models demonstrate that oncogenic KRAS and BRAF signalling directly increases tumour cell turnover and apoptotic/necrotic DNA release, providing a biological basis for their reliable detection in ctDNA. Notably, studies comparing tumour tissue and plasma in these models show high concordance of driver mutations, reinforcing ctDNA as a faithful surrogate of tumour genotype.

Additional ctDNA‐detected tumour suppressors and regulators – including PTEN, FBXW7, CDKN2A and RNF43 – have also been functionally interrogated using knockout or knockdown systems. PTEN loss in GI epithelial models results in sustained PI3K–AKT signalling, increased survival and resistance to apoptosis, conditions that promote persistent ctDNA release.^33^ FBXW7 knockout leads to stabilisation of oncogenic substrates such as MYC and Cyclin E, driving aggressive tumour behaviour and increased circulating tumour burden.^34^ RNF43 loss‐of‐function models demonstrate ligand‐dependent Wnt hypersensitivity, particularly in serrated and microsatellite instability (MSI)‐high tumours, explaining the enrichment of RNF43 alterations in ctDNA from these molecular subtypes.^35^

Collectively, these genetic perturbation studies establish a causal relationship between recurrent GI cancer driver alterations and tumour biology, providing strong mechanistic support for their detection in ctDNA. Importantly, they demonstrate that ctDNA alterations are not merely associative biomarkers, but direct molecular readouts of defined oncogenic pathways, tumour cell turnover and disease burden.

### 2.2 Circulating tumour cells

Circulating tumour cells (CTCs)are the intact cancer cells that detach from the tumour and enter the bloodstream. CTCs provide direct cellular material for analysis, enabling not only enumeration but also phenotypic and molecular characterisation (e.g., protein expression, single‐cell sequencing), which can inform on tumour heterogeneity, metastatic potential and mechanisms of drug resistance.^36^, ^37^ CTCs are extremely rare (∼1 per million blood cells) and survive only briefly in circulation.^38^, ^39^ Nevertheless, they can be captured and analysed for tumour DNA, RNA or protein markers. CTCs may be particularly informative for prognosis in GI cancers and for identifying patients at higher risk of metastasis, as their presence and quantity often correlate with disease burden and progression.^40^ Biomarker analysis is feasible on CTCs, including HER2, EGFR, PD‐L1 and Ki‐67, which can guide appropriately targeted therapy selection in colorectal and other GI cancers.^37^ However, detecting CTCs in early‐stage disease is challenging due to their low numbers, making detection technically challenging and sometimes unreliable for early diagnosis or screening. Current detection platforms have limitation – many rely on epithelial markers (e.g., EpCAM), potentially missing mesenchymal or stem‐like CTCs with high metastatic potential, and require specialised equipment and expertise.^40^, ^41^ Novel capture platforms (e.g., CellSearch®, size‐based microfilters, microfluidic chips) are being optimised to improve CTC.^42^, ^43^ CTC isolation and analysis are more labour intensive and costly compared with ctDNA, and standardisation across platforms remains a challenge, hindering routine clinical adoption.^44^

### 2.3 ExtracellulVs and exosomal RNA

Tumour cells release nano‐sized vesicles (exosomes and other EVs) into blood, carrying DNA, various RNAs and proteins reflective of the parent tumour.^45^ Exosomal cargo includes microRNAs (miRNAs) and other non‐coding RNAs that can serve as cancer biomarkers. Notably, exosomes carry surface proteins that can indicate their tissue of origin. This ‘zip code’ property has been exploited to identify pancreas‐derived exosomes in blood, enabling pancreatic cancer detection via exosomal miRNA profiles.^46^, ^47^ For example, exosomal glypican‐1 (a membrane protein) was reported to specifically mark pancreatic cancer exosomes and detect even early‐stage pancreatic tumours in a pilot study.^48^ Similarly, unique miRNA signatures in serum exosomes have been associated with gastric, oesophageal and colon cancers.^15^, ^49^ As an example, combining exosome isolation with RT‐PCR for specific miRNAs has enabled detection of Barrett's oesophagus and oesophageal cancer in blood, as shown in the multi‐centre EMERALD study which identified a miRNA panel distinguishing Barrett's/early oesophageal adenocarcinoma (EAC).^50^, ^51^ An emerging extension of EV‐ and miRNA‐based diagnostics is the analysis of miRNAs in exhaled breath condensate (EBC), a non‐blood liquid biopsy matrix that reflects airway and systemic cellular signalling.^52^, ^53^ Recent studies using next‐generation sequencing (NGS) have demonstrated the feasibility of detecting stable miRNA signatures in EBC, with robust analytical performance and minimal invasiveness.^52^, ^53^ Although most data are currently derived from lung cancer screening, the underlying methodology, capturing extracellular miRNAs released via vesicular pathways, has direct relevance to GI malignancies, particularly for aerodigestive tract tumours. As analytical sensitivity improves, EBC‐based miRNA profiling may complement blood‐based EV assays and expand the spectrum of minimally invasive cancer detection strategies. While exosome isolation and analysis technologies are still maturing, EV‐based biomarkers hold promise for multi‐analyte early detection assays.

### 2.4 Tumour proteins and other circulating biomolecules

Classic serum tumour markers (proteins) such as carcinoembryonic antigen (CEA) in colorectal cancer (CRC) or CA19‐9 in pancreatic cancer are detectable via blood tests.^54^ However, their sensitivity for early‐stage disease is poor (e.g., 20–25% of all patients with pancreatic cancer are non‐shedders of CA19‐9),^55^ and many slow‐growing or small tumours do not produce elevated marker levels until they are larger, resulting in frequent false negatives.^56^ For example, in asymptomatic individuals, the sensitivity of CEA and CA19‐9 for GI and whole‐body cancer screening is only 7–11%.^57^ CA19‐9 sensitivity for pancreatic cancer is about 72–80% in symptomatic patients, but this drops substantially in early‐stage or screening settings.^56^, ^58^ These markers are not cancer specific. CA19‐9 and CEA can be elevated in benign conditions such as pancreatitis, cholestasis, cirrhosis, inflammatory bowel disease and even with lifestyle factors like smoking.^59^ This leads to false positives and diagnostic uncertainty, as elevations may mimic malignancy but actually reflect non‐cancerous pathology. About 5–10% of the population are Lewis antigen negative and cannot produce CA19‐9, so their levels remain normal even in the presence of pancreatic cancer, resulting in false negatives.^60^ New proteomics approaches are identifying panels of protein markers that, in combination, improve early detection. For instance, the multi‐protein panel used in the CancerSEEK test included CA125, CEA, CA19‐9 and others to broaden detection across multiple GI tumours.^10^ Beyond proteins, other exploratory liquid biopsy analytes include circulating tumour‐derived platelets (platelets ‘educated’ by tumours show distinct RNA profiles; TEPs) and metabolites.^61^ These are in early research stages but could augment DNA/RNA‐based detection.

## 3 ADVANCES IN DETECTION TECHNOLOGIES

**FIGURE 2:**
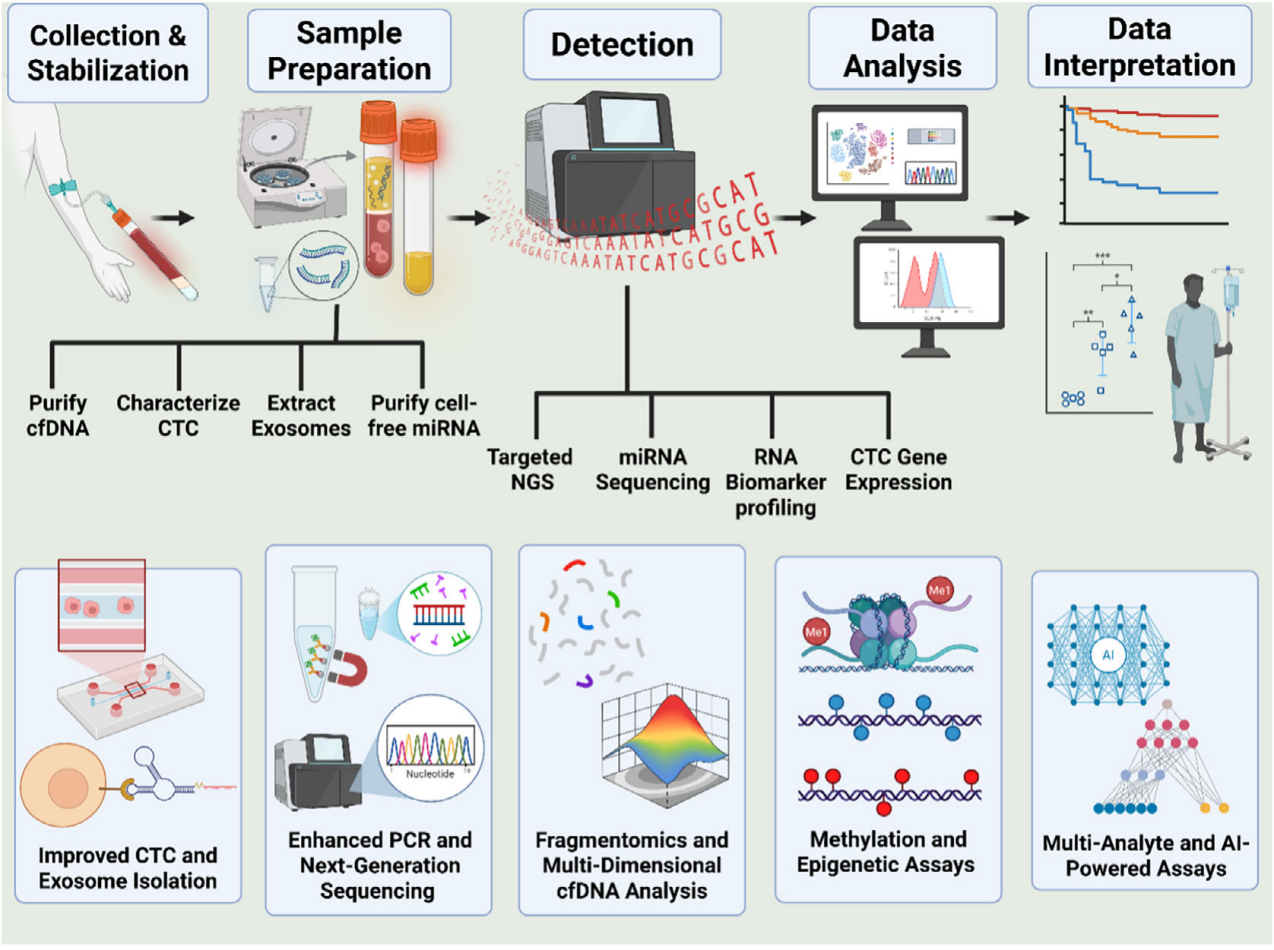
Workflow of liquid biopsy for cancer detection. This schematic illustrates the key steps involved in liquid biopsy, from sample collection and stabilisation to data interpretation. It includes collection of blood, separation of plasma and preparation of various analytes such as circulating tumour DNA (cfDNA), circulating tumour cells (CTCs), extracellular vesicles (EVs) and cell‐free microRNA (miRNA). These analytes are then processed using techniques like targeted next‐generation sequencing (NGS), miRNA sequencing, RNA biomarker profiling and CTC gene expression analysis. Finally, the collected data undergo analysis and interpretation for cancer detection. *Abbreviations*: cfDNA, cell‐free DNA; CTC, circulating tumour cell; EVs, extracellular vesicles; miRNA, microRNA; NGS, next‐generation sequencing; RNA, ribonucleic acid; PCR, polymerase chain reaction liquid biopsy applications by GI cancer type.

Detecting tiny signals from incipient tumours requires progressive assay technologies. Recent innovations have greatly enhanced the sensitivity and accuracy of liquid biopsies for early cancer detection (Figure 2).

### 3.1 Enhanced PCR and NGS

Traditional PCR can miss very low‐frequency mutations, so digital PCR methods like droplet digital PCR (ddPCR) and BEAMing (beads, emulsion, amplification, magnetics) were developed to detect one mutant molecule among thousands of wild‐type.^62^ These have been used, for example, to detect KRAS mutations in plasma for pancreatic cancer. NGS technologies now enable broad sequencing of dozens to hundreds of genes in ctDNA. Techniques such as tagged‐amplicon deep sequencing (TAm‐Seq) and CAPP‐Seq (Cancer Personalised Profiling by deep sequencing) incorporate unique molecular identifiers and error‐suppression to achieve high sensitivity.^63^, ^64^ These methods can detect mutant allele fractions below.1%, crucial for early‐stage tumours with minimal ctDNA. Targeted NGS panels (covering common GI cancer mutations in genes like *TP53*, *APC*, *RAS*) and even whole‐genome sequencing of cfDNA are being applied in research settings to capture myriad tumour signals.

### 3.2 Methylation and epigenomic assays

Tumour‐specific DNA methylation changes are often more consistent than mutations in early neoplasia.^65^ Several blood tests exploit methylation markers to improve sensitivity. For example, the United States Food and Drug Administration (US FDA)‐approved Epi proColon test for CRC detects methylated *SEPT9* gene in plasma DNA.^66^ Emerging assays perform bisulphite sequencing of cfDNA at numerous CpG sites to detect cancer‐specific methylation patterns. Notably, the GRAIL Galleri® test uses targeted methylation sequencing and machine learning to detect a methylation ‘signature’ of cancer and even predict the tissue of origin.^67^ In GI cancers, research has identified panels of hypermethylated genes detectable in blood for early detection – for example,  methylation of certain genes in gastric cancer.^68^ cfDNA assays utilising these epigenetic markers have demonstrated high sensitivity and specificity in initial studies. Epigenetic assays can also address issues like clonal haematopoiesis by focusing on tumour‐specific methylation sites. Beyond 5‐methylcytosine, mapping of 5‐hydroxymethylcytosine (5hmC) in cfDNA via specialised techniques (e.g., 5hmC‐Seal) has shown promise for early hepatocellular carcinoma (HCC) detection.^69^ Using a retrievable sponge‐capsule device to detect oesophageal cancer precursors, researchers identified a three‐gene DNA methylation panel (USP44, TBC1D30 and NELL1) that diagnosed Barrett's oesophagus, high‐grade dysplasia and EAC with high accuracy (AUCs up to.969) in both training and test sets. This method provides a practical and accessible screening tool for early detection of these high‐risk oesophageal conditions.^70^

### 3.3 Fragmentomics and multi‐dimensional cfDNA analysis

Tumour‐derived cfDNA often exhibits distinct fragmentation patterns – typically shorter fragment lengths and specific nucleosome footprints. Advanced whole‐genome sequencing of cfDNA can capture fragment size distributions, nucleosome occupancy patterns and copy number variations.^71^ By integrating these features with mutation data, machine learning models can dramatically improve detection accuracy. A recent study in early gastric cancer exemplified this ‘multi‐dimensional’ approach: by combining four cfDNA features (fragment sizes, nucleosome coverage, CNVs and mutations), researchers developed an ensemble model for detecting stage II–III gastric cancer.^72^ This far exceeds the performance of any single marker and underscores how AI‐driven analysis of big genomic data can boost early detection.

### 3.4 Improved CTC and exosome isolation

On the CTC front, newer microfluidic devices and antibody‐independent capture methods are increasing yield. For instance, the US FDA‐cleared CellSearch system (EpCAM‐based capture) is used for CRC prognosis, but misses CTCs that have lost epithelial markers. Size‐based platforms like Parsortix™ enrich CTCs by filtration and can capture both epithelial and mesenchymal tumour cells.^73^ Immunostaining strategies and negative depletion of blood cells are also employed. To detect microvesicles and exosomes, ultracentrifugation is being supplemented by microfluidic exosome chips and nanoparticle tagging to selectively isolate tumour exosomes. These allow sensitive downstream analysis of exosomal DNA or RNA. Continued engineering advances are expected to improve the recovery of these rare circulating markers.

### 3.5 Multi‐analyte and Artificial intelligence (AI)‐powered assays

Given that single biomarkers often lack adequate sensitivity or specificity, many current efforts focus on integrating multiple analytes and using machine learning to interpret complex patterns. (AI) plays a central role in contemporary multi‐analyte liquid biopsy platforms by enabling integration of heterogeneous molecular data, including genomic, epigenomic, transcriptomic and proteomic features.^74^. Machine‐learning algorithms are employed for feature extraction, dimensionality reduction and signal denoising, allowing discrimination of tumour‐associated patterns from background biological noise. Supervised and deep‐learning models, including ensemble classifiers and neural networks, are increasingly used to generate composite cancer probability scores and tissue‐of‐origin predictions. These approaches are particularly valuable in early‐stage disease, where individual biomarkers may fall below conventional detection thresholds but collectively yield a robust diagnostic signal.

The clinical utility of this multi‐analyte, machine‐learning‐driven paradigm has been demonstrated in prospective studies such as the DETECT‐A trial, which utilised the CancerSEEK MCED blood test to analyse ctDNA and tumour‐associated proteins, coupled with PET‐CT imaging, to identify multiple cancer types, including ovarian, thyroid, uterine, breast, colorectal and lung cancers. After a median follow‐up of 4.4 years, 50% of participants whose cancers were detected by CancerSEEK were alive and cancer‐free. Notably, over half of these participants (54%) had cancers for which there are no standard‐of‐care screening modalities. Furthermore, 100% of stage I or II participants who received treatment and 86% of surgically treated participants were alive and cancer‐free, suggesting that early detection via CancerSEEK may facilitate curative‐intent treatments and improve clinical outcomes.^75^ Machine learning models also underpin multi‐cancer early detection (MCED) tests like Galleri®, which analyses methylation patterns across the genome to signal the presence of any of 50 cancer types with high specificity. For GI cancers specifically, researchers are exploring combined panels of DNA + RNA + protein.^76^ In pancreatic cancer, for example, one approach measured both circulating miRNAs and the CA19‐9 protein: this combined test accurately identified 97% of early‐stage pancreatic cancers in a large study.^77^ Such multi‐analyte strategies harness complementary biomarkers and use AI algorithms for classification, greatly enhancing detection performance for early tumours.

## 4 MCED AND SINGLE CANCER ASSAYS

Recent advancements in liquid biopsy technology have introduced two distinct approaches for cancer detection: single‐cancer assays and emerging MCED tests. Single‐cancer liquid biopsies have found clinical utility for specific populations, such as the use of the US FDA‐approved Epi proColon test as a screening option for adults at average risk for colorectal cancer who are unwilling or unable to undergo traditional screening approaches.^102^ In other GI cancers, single‐target assays are often used to detect specific mutations (e.g., *KRAS* mutation in pancreatic cancer ctDNA) for monitoring or guiding therapy rather than general screening.^103^

In contrast, MCED tests are a new paradigm designed for screening apparently healthy, asymptomatic individuals for multiple cancer types simultaneously, many of which (like pancreatic or ovarian cancer) lack standard screening pathways.^104^ The primary goal of MCED tests is to reduce overall cancer mortality by shifting the diagnosis of a wide spectrum of cancers to earlier, more treatable stages. A recent prospective validation study from the PATHFINDER II trial (NCT05155605) further informs the clinical utility of MCED assays, providing real‐world evidence on diagnostic yield, downstream testing and the feasibility of integrating MCED screening into routine clinical practice. While performance varies by cancer type, a high priority is placed on achieving extremely high specificity (often > 98%) to minimise the burden of false positives in a low‐prevalence screening population, which would otherwise necessitate complex and potentially invasive diagnostic workups. The positive predictive value (PPV) can still be a challenge, with studies reporting PPVs below 50%, meaning a significant portion of positive results do not lead to a cancer diagnosis after an extensive workup.^105^

The advantage of MCED tests is their broad, minimally invasive nature and ability to detect cancers that currently lack screening guidelines, potentially improving adherence and reducing barriers to screening. The main drawbacks are their generally lower sensitivity for early‐stage cancers and the potential for patient anxiety and complex diagnostic workups following a positive result. In contrast, single‐cancer liquid biopsies offer superior sensitivity and specificity for their specific target cancer within specific clinical contexts and are supported by more extensive clinical evidence for guiding treatment and monitoring. However, they address only a limited number of cancer types, leaving many cancers undetected until a later, more symptomatic stage. Therefore, while MCED tests are a promising tool to fill critical screening gaps, they are currently recommended as a complement, not a replacement, for existing screening methods.

## 5 LIQUID BIOPSY APPLICATIONS BY GI CANCER TYPE

### 5.1 Colorectal cancer

CRC is a model case for liquid biopsy screening, as it is common and has well‐defined early interventions (polyp removal prevents cancer). The first blood‐based CRC screening test, Epi proColon, detects methylated *SEPT9* DNA in plasma. It received US FDA approval in 2016 offers a screening avenue for patients unable or unwilling to undergo other screening methods. While convenient, its sensitivity for early‐stage or precancerous lesions is modest, and guidelines still favour stool‐based tests and colonoscopy. However, next‐generation blood tests are rapidly advancing.^1^ In 2024, the Guardant Shield test (cfDNA‐based) became the first US FDA‐approved blood test for average‐risk CRC screening.^106^ In a 20 000‐patient trial (ECLIPSE), Shield detected 83% of CRCs.^106^ Nonetheless, having an accurate blood test could increase screening uptake among those averse to colonoscopy. Other assays in development include Guardant's earlier LUNAR‐2 panel and various multi‐marker tests. Multi‐omic approaches measuring cfDNA mutations, methylation and even microbiome signals from blood are being explored to further boost sensitivity for early CRC and even advanced adenomas. In practice, any positive blood test must be followed by diagnostic colonoscopy. As blood assays improve, they may serve as an initial screen to identify individuals who need colonoscopy, thereby widening the net of CRC screening.

Increasingly, multi‐omic liquid biopsy approaches are being developed to overcome the limited sensitivity of single‐analyte assays for early‐stage CRC. By integrating cfDNA mutations, genome‐wide methylation patterns, fragmentomic features and RNA‐based biomarkers, these platforms generate composite risk scores that outperform individual markers.^107^ Such integrative strategies leverage complementary biological signals associated with tumour initiation and progression, enabling improved early‐stage detection, advanced adenoma identification and personalised risk stratification. As highlighted in recent analyses, multi‐omic frameworks are likely to define the next generation of CRC screening assays, particularly when combined with machine‐learning‐based classification models.

### 5.2 Gastric cancer

Gastric cancer often presents late, especially in Western countries without routine endoscopic screening.^108^ Liquid biopsy approaches under investigation could enable population screening for this deadly cancer.^109^ ctDNA is detectable in many gastric cancer patients, and studies show it can complement endoscopy. For example, methylation of certain tumour suppressor genes in plasma ctDNA (such as *PCDH10* or *RASSF1A*) distinguished gastric cancer patients from controls with promising accuracy.^110^, ^111^ Comprehensive cfDNA methylation profiling has even suggested that different GI tumour types have distinct methylation ‘fingerprints’ in blood.^81^ Beyond DNA, researchers have identified miRNA panels that are elevated in early gastric cancer patients’ plasma; some of these panels approach sensitivities ∼80% in initial trials.^112^, ^113^ An approach has been the use of fragmentomics and whole‐genome cfDNA analysis in gastric cancer: a 2024 study by Yu et al. developed a cfDNA‐based early detection model that achieved 88.2% sensitivity and 92.1% specificity for gastric cancer by integrating fragment size patterns, nucleosome occupancy, CNVs and mutations.^114^ This multi‐dimensional liquid biopsy significantly outperformed single biomarkers and even simulated to compare favourably with endoscopy in a screening context. Such data are highly encouraging, though larger validation is needed. Challenges remain because early‐stage gastric tumours have limited DNA or CTCs. Combining liquid biopsy with imaging or gastroscopy might further improve sensitivity.^115^ While no blood test for gastric cancer is yet in clinical use, a pipeline of assays targeting ctDNA mutations like *TP53*, methylation signatures and exosomal RNAs are in clinical trials for early gastric cancer detection.^15^ These could be an initiation for minimally invasive screening in high‐risk populations.

### 5.3 Oesophageal cancer

Early detection of oesophageal cancer is difficult – EAC often arises from Barrett's oesophagus (BE), a precancerous condition and oesophageal squamous cell carcinoma (ESCC) has high incidence in certain geographical regions.^116^ Liquid biopsy research is beginning to tackle this. Plasma ctDNA levels in oesophageal cancer correlate with tumour stage and can signal MRD after treatment, but detecting de novo early‐stage disease via ctDNA is challenging due to low scale.^117^ Nonetheless, recent advances show promise: the EMERALD study (2025) evaluated a blood miRNA assay in 792 individuals and was able to identify patients with BE and even those with dysplasia or EAC with high accuracy.^86^ The EMERALD study developed and validated a novel six‐miRNA blood test that shows promise for the early detection of EAC and its precursor lesion, BE.^86^ Specifically, a panel of circulating miRNAs was developed and externally validated to differentiate BE/EAC cases from controls, indicating a potential screening tool for at‐risk patients.^118^ Other approaches include assays for tumour‐specific methylated DNA in plasma – for example, methylation of the *NELL1* gene has been reported as a biomarker for BE/EAC detection.^119^ In ESCC, unique methylation markers and somatic mutations like in *TP53* or *NOTCH1* can be found in cfDNA,^120^ these are being studied especially in regions with mass screening programs. While no liquid biopsy test for oesophageal cancer is yet in practice, the field is moving towards combined biomarker strategies (ctDNA + miRNA) and even swallowed ‘sponge’ devices that collect oesophageal cells for analysis of molecular markers as a complementary minimally invasive approach.^121^ Liquid biopsy may ultimately enable less invasive surveillance of Barrett's patients and earlier diagnosis of oesophageal malignancy, but further validation in large cohorts is needed.

### 5.4 Pancreatic cancer

Pancreatic ductal adenocarcinoma (PDAC) is notorious for late diagnosis and poor survival, underscoring the need for an early detection test.^122^ However, screening the general population is impractical due to the cancer's low incidence. Liquid biopsy offers hope, especially for high‐risk groups (family history, genetic syndromes, new‐onset diabetes, etc.). ctDNA is present in a fraction of early PDAC patients – in one early study, PCR detected *KRAS* mutations in blood of pancreatic cancer patients, even mirroring the mutations in tumour tissue.^123^ However, ctDNA sensitivity for small, localised PDAC remains limited (tumours often deep‐seated). Hence, multi‐biomarker blood tests are being explored. A notable recent advancement is a miRNA‐based assay, where researchers developed a test analysing a panel of miRNAs (both free and exosome‐contained) that, when combined with serum CA19‐9, correctly identified 97% of patients with stage I–II pancreatic cancer in a 1000‐patient study.^124^, ^125^ This two‐component approach leverages miRNA abundance and stability, plus the established (but not very sensitive) CA19‐9 marker, achieving a level of accuracy unheard‐of for PDAC. Another promising biomarker is glypican‐1 (GPC1) on tumour‐derived exosomes – a 2015 study in *Nature* reported that GPC1+ circulating exosomes distinguished early pancreatic cancer, suggesting near 100% sensitivity and specificity in a small cohort.^48^ While these results need broader validation, it spurred interest in exosomal protein markers for PDAC. Additionally, various blood protein panels (e.g., combining CA19‐9 with novel markers like TIMP1, LRG1) have been evaluated with sensitivities in the 70–85% range for early PDAC when used as a panel. Because single markers are insufficient, multianalyte panels are favoured: a recent comprehensive review emphasises that combining circulating DNA, various RNAs and proteins yields better sensitivity for PDAC than any single biomarker. For example, the ongoing PanCAN ‘DETECT’ study and the PRECEDE consortium are testing such multi‐marker blood tests in high‐risk individuals (e.g., those with familial risk or new diabetes) to catch PDAC earlier. While no liquid biopsy test for pancreatic cancer is yet approved for routine screening, these investigative trials and assays (some in the clinical laboratory as LDTs) mark significant steps towards a viable early detection tool for this devastating cancer.^123^

### 5.5 HCC (liver cancer)

HCC often develops in the setting of chronic liver disease (hepatitis or cirrhosis), and surveillance in high‐risk patients is recommended. Current standard surveillance with ultrasound and the protein marker Alpha Fetoprotein (AFP) has suboptimal sensitivity for early HCC. Liquid biopsy approaches are poised to enhance early HCC detection.^126^ Patients with liver cancer actually tend to have higher total cfDNA levels than other cancers, due to circumventing first pass metabolism, making HCC a good candidate for cfDNA‐based tests. Plasma *ctDNA* in HCC can reveal mutations (e.g., *TP53*, *CTNNB1*) or viral integrations, but early‐stage tumours may not show these signals consistently.^127^ The focus has thus shifted to cfDNA methylation markers and multi‐analyte tests. One of the most advanced is the HelioLiver test, a blood assay that combines cfDNA methylation signatures with protein markers (AFP, AFP‐L3%, des‐γ carboxyprothrombin [DCP]) and patient factors. In a blinded multi‐centre validation, HelioLiver detected early‐stage (stage I/II) HCC with 76% sensitivity at 91% specificity, significantly outperforming AFP alone or the GALAD score (computed from the linear combination of demographic and lab‐based factors; Gender, Age, Level of AFP‐L3, AFP level and DCP level).^128^ This multi‐parametric test is now offered as a Laboratory‐Developed test(LDT) for high‐risk patients and is being integrated into surveillance studies. Other groups have developed ctDNA methylation panels for HCC; for example, the cfDNA 5hmC mapping by Cai et al. achieved an AUC around .94 for early HCC versus cirrhosis. Additionally, the GRAIL multi‐cancer test has reported detection of asymptomatic HCC in some cases via methylation signals.^129^ Aside from DNA, circulating miRNAs (e.g., the miR‐122 family) has been proposed as early HCC markers, and metabolomic profiles of plasma are being examined.^130^ Urine‐based assays are also under exploration – for example, urine cfDNA methylation has shown potential for liver cancer detection.^131^ With multiple approaches in play, HCC might soon see improved surveillance protocols: for instance, trials are underway comparing blood DNA tests versus ultrasound in high‐risk patients.^132^ The goal is to catch HCC at curative stages more reliably than current methods, and liquid biopsies appear to hold the key to that improvement.

### 5.6 Cholangiocarcinoma (biliary tract cancer)

Cholangiocarcinoma, including cancers of the bile ducts, is relatively rare but deadly, often arising in the context of primary sclerosing cholangitis (PSC) or liver fluke infection.^133^ Early detection is exceedingly difficult as there are no established screening methods for at‐risk groups,^133^ and patients are recommended to undergo low sensitivity imaging modalities. Liquid biopsy research is in early stages here. Plasma ctDNA has shown utility in diagnosing cholangiocarcinoma in symptomatic patients – for example, sequencing can detect characteristic mutations like *IDH1* or *FGFR2* fusions that confirm the presence of malignancy.^134^, ^135^ In a small series, detectable ctDNA was associated with poorer outcomes in biliary cancer patients. For early, asymptomatic detection, one strategy is analysing bile fluid (obtained during endoscopy) for tumour DNA or exfoliated CTCs; this can sometimes catch cholangiocarcinoma earlier than blood assays given the localised nature of it.^136^ Some studies have identified methylated DNA markers in bile that differentiate malignant from benign biliary strictures with high accuracy. Still, in blood, the very low tumour burden in early cholangiocarcinoma limits detection capability. Researchers are examining combinations of cfDNA mutations and protein markers (e.g., CA19‐9, IL‐6) for earlier signals.^137^, ^138^, ^139^ No other avenue is monitoring high‐risk individuals (like PSC patients) with periodic liquid biopsies to detect any rising tumour‐associated mutation or methylation signature. Though this approach remains investigational, it could supplement imaging and flag incipient cholangiocarcinoma. Overall, liquid biopsy for biliary cancers lags other GI cancers in development, but as sequencing sensitivity improves and unique biomarkers are found, it may become feasible to intercept these cancers earlier than is currently possible.

## 6 CLINICAL TRANSLATION AND ONGOING TRIALS

Tremendous progress has been made in translating liquid biopsy from bench to bedside in GI oncology. Several assays have moved into clinical practice or late‐stage trials, heralding a new era of minimally invasive cancer screening. In CRC, the pattern is shifting now that blood tests like Shield (Guardant) have shown efficacy in large trials and gained US FDA approval.^140^ The challenge ahead is to integrate such tests into screening programs: studies are ongoing (e.g., the randomised PATHFINDER studies using the Galleri MCED test) to determine how blood‐based multi‐cancer screening can be implemented and whether it reduces mortality.^141^ For single‐cancer focused tests, trials like ECLIPSE (CRC) and prospective cohorts for HCC surveillance (using HelioLiver or others) are providing critical real‐world performance data. Table 2 highlights a selection of liquid biopsy assays and trials in GI cancers, illustrating the breadth of translational efforts.

**TABLE 2:** Liquid biopsy approaches for early detection of GI cancers: Key biomarkers, technologies and current status.

GI cancer	Key liquid biopsy biomarkers (examples)	Detection technologies (assay)	Early detection performance/status	References
Colorectal	Methylated ctDNA: SEPT9 (Epi proColon test), BMP3, NDRG4 (cologuard test). cfDNA fragmentation: genome‐wide fragment analysis. miRNAs: miR‐21, miR‐29a	NGS: wide genomic and exome sequencing for mutations. Quantitative PCR (qPCR): to detect specific methylated DNA markers. ddPCR: highly sensitive for low‐concentration ctDNA.	US FDA‐approved tests: Epi proColon (SEPT9) and Cologuard (stool DNA) demonstrate clinical utility. High sensitivity studies: some studies show ctDNA methylation can detect CRC up to 2 years before diagnosis in high‐risk patients, with over 40% sensitivity and 85% specificity. Emerging tests: efforts are focused on improving sensitivity for early‐stage and pre‐cancerous lesions, where ctDNA concentration is low.	^6^, ^78^ ^,^ ^80^
Gastric	Methylated cfDNA: cg10673833, DOCK10, CABIN1, XAF1. Exosomal long non‐coding RNAs: GClnc1. Multi‐omics signatures: combination of cfDNA, ctDNA and exosomal RNA markers.	NGS): genome‐wide methylation analysis. qPCR: multiplex assays like the SpecGastro test. EV analysis: for exosomal RNA markers.	High diagnostic accuracy: multiplex qPCR assays like SpecGastro have shown over 75% sensitivity and 89% specificity for detecting GI cancers, including gastric cancer. Promising biomarkers: EV‐derived GClnc1 was validated in multiple cohorts with AUCs over.88 for early‐stage detection. Ongoing clinical trials: numerous trials focus on identifying sensitive and specific biomarkers for early detection and MRD.	^81^ ^,^ ^85^
Oesophageal	ctDNA: Tumour‐specific mutations in genes like TP53, CDKN2A, SMAD4 and NOTCH1 • DNA methylation markers, such as SEPTIN9, TFPI2, FHIT, RASSF1A, SHOX2, EPO and RNF180. miRNAs: Specific panels of circulating miRNAs, including overexpression of miR‐106b, miR‐146a, miR‐15a, miR‐18a, miR‐21 and miR‐93. Other examples: miR‐1972, miR‐4274, miR‐4701‐3p, miR‐6126, miR‐1268a and miR‐4505. CTCs: tumour cells from the primary tumour. Often characterised by epithelial cell adhesion molecule (EpCAM) or other markers, though EMT can cause low EpCAM expression. Other biomarkers: long non‐coding RNAs (lncRNAs): examples include *lnc‐POU3F3*. Metabolites: profiling of molecules in blood. Autoantibodies: antibodies against tumour antigens, such as p53, p16, HSP70 and c‐Myc.	ddPCR: a highly sensitive method for quantifying low‐concentration ctDNA. NGS: used for targeted or whole‐exome sequencing to identify a broader range of mutations. qPCR: used in multiplex assays, such as the SpecGastro test, to detect specific methylation biomarkers microarray and qPCR: standard methods for miRNA analysis. ddPCR and NGS: increasingly used for comprehensive miRNA profiling. Immuno‐magnetic enrichment: uses EpCAM antibodies to capture CTCs (e.g., CellSearch system). Physical filtration: isolates CTCs based on size and rigidity (e.g., ISET system). Microfluidic devices: capture CTCs in controlled flow conditions. Quantitative PCR (qPCR): to detect lncRNAs. Mass spectrometry (MS): used in metabolomics for profiling metabolites. ELISA: for measuring levels of circulating autoantibodies.	In development: performance is limited by low ctDNA concentrations in early‐stage disease, leading to moderate sensitivity. Variable sensitivity: a 2022 meta‐analysis reported 71.0% pooled sensitivity and 98.6% specificity for diagnosing EC. Staging differences: one study found a ctDNA positive rate of 14.3% in stage I ESCC compared with 85.2% in stage II+. Early results: a recent study found that a methylation‐based cfDNA assay achieved 84.7% overall sensitivity, with 69.5% for early‐stage (I/II) EC. Promising results: one miRNA signature, EMERALD, detected EAC with 84.0% sensitivity and 90.5% specificity. Potential for screening: the EMERALD test also detected BE, a precursor to EAC, with 80.6% sensitivity. High accuracy: the EMERALD signature showed an AUC of 91.9%. Promising results: one miRNA signature, EMERALD, detected EAC with 84.0% sensitivity and 90.5% specificity. Potential for screening: the EMERALD test also detected BE, a precursor to EAC, with 80.6% sensitivity. High accuracy: the EMERALD signature showed an AUC of 91.9%.	^86^ ^,^ ^93^
Pancreatic	Exosomal microRNAs: multi‐panel microRNA signatures. Methylated DNA markers (MDMs): in plasma and pancreatic juice. Multi‐marker panels: combinations of cfDNA, microRNAs and CA19‐9.	High‐throughput sequencing: for identifying multi‐marker signatures. qPCR: to measure specific methylated DNA markers. Machine learning: used to develop and validate multi‐marker detection signatures.	High accuracy potential: investigational multi‐marker blood tests show high accuracy (up to 97%) for early‐stage pancreatic cancer when combined with CA19‐9. Addressing limitations of current standard: tests are designed to overcome the low sensitivity of traditional marker CA19‐9 in early stages. Promising approach: exosome‐based signatures are effective in detecting early‐stage disease and have been validated in multi‐ethnic cohorts. Early stages of research: these biomarkers show potential but require more large‐scale validation studies to confirm their role in early detection. Panels improve performance: combining multiple markers, such as lncRNA panels, shows promise for enhanced accuracy.	^94^ ^,^ ^96^
Hepatocellular (HCC)	Methylated cfDNA: SEPT9, multi‐gene panels (e.g., APC, RASSF1A, GSTP1). cfDNA fragmentation: Genome‐wide fragmentation profiles. Exosomal RNAs: circRNA, mRNA (e.g., GPC‐3 mRNA). cfDNA 5‐hydroxymethylcytosine: multi‐feature signatures.	Enzymatic methyl sequencing (EM‐seq): for identifying epigenetic variants. (NGS: for multi‐feature analysis (HIFI method). ddPCR: to quantify specific mutations or methylation.	High diagnostic performance: assays combining cfDNA methylation, fragmentation and other features have shown high sensitivity (over 95%) and specificity for differentiating HCC from liver cirrhosis. Superior to standard methods: some liquid biopsy techniques show higher sensitivity (85%+) for early HCC compared with standard ultrasound with AFP. Standardisation needed: future efforts require standardisation of collection and detection methods for improved credibility.	^97^ ^,^ ^99^
Cholangiocarcinoma	Bile ctdna: KRAS, TP53 mutations. Serum/bile non‐coding RNAs: microRNAs (E.G., Mirna‐21). EV cargo: exosomal protein biomarkers.	NGS: for identifying genomic alterations. CRISPR/Cas systems: emerging technologies for rapid, specific biomarker detection. ELISA/immunoassays: For protein biomarkers in serum EVs.	Bile shows strong concordance: bile‐based liquid biopsy shows strong concordance with tissue biopsies for mutation detection, suggesting it could be a viable alternative. High‐risk screening potential: liquid biopsy tools could help identify high‐risk populations for targeted screening. Future focus: the integration of multi‐omics approaches is key to enhancing diagnostic accuracy.	^100^, ^101^

## 7 CURRENT LANDSCAPE OF LIQUID BIOPSY RESEARCH FOR EARLY CANCER DETECTION

The landscape of liquid biopsy for early detection of GI cancers is dynamically evolving, driven by extensive research into various circulating biomarkers (Table 3). Currently, a significant number of these studies are observational prospective cohorts and observational retrospective case–control trials, with some interventional prospective and randomised trials also contributing, all designed to identify and validate minimally invasive methods for early cancer detection. These trials primarily investigate how different biomarker types in blood can offer an earlier diagnostic window, often before clinical symptoms appear.

**TABLE 3:** Overview of ongoing liquid biopsy clinical trials for early detection of gastrointestinal cancers: biomarkers, technologies and status.

Cancer type	Registry ID/year, country	Study aim	Study design	Sample size	Key liquid biopsy biomarkers	Sample type	Detection technology assays
	NCT06342440, started 2020 United States of America	Develop a sensitive, specific and cost‐effective blood assay for early detection of colorectal adenomas and cancer	Observational retrospective case control	2000	Diagnostic test: DENEB, a panel of circulating microRNA (cf‐miRNA and exo‐miRNA)	Blood	Genome‐wide miRNA profiling → machine learning‐based biomarker selection → qPCR‐based validation; combined cf‐miRNA & exo‐miRNA signature (DENEB)
cr	NCT06342401, started 2023 United States of America	Develop and validate a liquid biopsy assay for the detection of early‐onset colorectal cancer (<50 years of age)	Observational retrospective case control	400	Diagnostic test: ENCODE, a panel of circulating microRNA (cf‐miRNA and exo‐miRNA)	Blood	Small RNA‐Seq profiling → machine learning‐based biomarker selection → validation on independent early onsetCRC vs. control cohort
Colorectal	NCT06258434, started 2024 China	Sensitivity and specificity of the multiomics colorectal cancer screening test (CRC‐Appareo) with comparison to colonoscopy	Observational prospective cohort	1000	cfDNA methylation, fragment patterns and copy number variations	Blood	Reduced representation bisulphite sequencing and using machine learning to accurately assess risk of CRC
Colorectal	NCT06991452, started 2025 China	Diagnostic performance of a multi‐omics approach and serum tumour markers for early CRC detection and recurrence	Observational prospective cohort	1200	5hmC biomarkers in cfDNA (oxidised derivative of 5‐methylcytosine, reflecting DNA methylation dynamics)	Blood	Genome‐wide 5hmC profiling using NGS; generating 5hmc signatures for CRC detection using machine learning models
Colorectal	NCT06989814, started 2025 Netherlands	Develop a liquid biopsy test that can detect CRC in early stages in patients with lynch syndrome	Interventional prospective case control	50	Tumour agnostic ctDNA characteristics	Blood	Multi‐omics approach for analysing ctDNA
Colorectal	NCT04792684, 2020 United States of America	Optimise the biomarker panel and evaluate the performance of cfDNA marker panel for CRC and advanced adenoma detection	Observational prospective case control	1300	Methylated cfDNA	Blood	NGS to profile cfDNA methylation changes to establish a proprietary panel of biomarkers combined into targeted assays
Colorectal	NCT07035691, started 2025 United Kingdom	Evaluate if a blood test for circulating progastrin (hPG80) and transposable elements (TEs) can accurately predict CRC or polyps in adult patients referred to the 2‐week wait (2WW) or Straight to Test (STT) pathways for suspected lower GI cancer	Observational prospective cohort	582	hPG80 and tTEs including transcript signatures and epigenetic modifications of DNA	Blood	Enzyme‐linked immunosorbent assay (ELISA) for hPG80; RNA sequencing and nanopore DNA sequencing for TEs
Colorectal	NCT05648240, started 2023 France	Proof‐of‐concept study to investigate if liquid biopsy can be used to detect biomarkers of tumour spread in patients with intestinal polyps	Observational prospective cohort	120	CTCs and circulating protein biomarkers including M‐CSF, GM‐CSF, CCL‐2, CXCL‐1, TIMP‐1 and SDF‐1	Blood	ANGLE Parsortix™ system for number of CTCs and ELISA for protein biomarkers
Colorectal	NCT06220617, started 2024 China	Develop a liquid biopsy prediction model for early CRC detection using multi‐omics markers	Observational prospective case control	3600	cfDNA, miRNA and other cancer‐specific markers	Blood	NGS for sequencing and analysing cfDNA and miRNA and machine learning for prediction model with external validation
Colorectal	NCT07090291, started 2025 China	Compare colonoscopy plus fecal immunochemical test (FIT) and FIT plus blood test for CRC screening	Open label cluster interventional randomised parallel group trial	62200	Methylated cfDNA	Blood	Specific technique not disclosed
Colorectal	NCT06955767, started 2025 France	Determine the cut‐off value for MMP‐14 for colorectal screening and evaluate the performance of the associated cut‐off value	Open label interventional cross‐sectional	650	MMP‐14 exosomal protein	Blood	ELISA
Colorectal	NCT06703632, started 2025 Portugal and Spain	Evaluate PreveCol's® efficiency as a second‐line method for detecting CRC and advanced adenomas after a positive fecal occult blood test (FOBT) result	Open label interventional single group prospective cohort	4538	11 proteins and 10 miRNAs	Blood	Biomarkers are measured using RT‐qPCR (for miRNAs) and ELISA/CLIA (for proteins). Machine learning techniques used to develop an ensemble algorithm to detect CRC based on proteins, miRNA and clinical features.
Colorectal	NCT06059963, started 2024 United States of America	Evaluate the performance characteristics of Signal‐C™ a plasma cfDNA test, to detect colorectal cancer and advanced precancerous lesions (APL)	Observational prospective cohort	15000	cfDNA	Blood	Uses targeted hybridisation‐based NGS with machine learning algorithm for detection and combination of methylation and fragmentation associated DNA marker regions
Colorectal	NCT07168876, started 2024 Argentina	Assess the performance of Delta‐HLD Epiliquid's proprietary liquid biopsy technology, for CRC detection	Observational cross sectional	3200	cfDNA, Delta‐HLD, Epiliquid's test	Blood	Specific technique not disclosed
Colorectal	NCT06880055, started 2025 United States of America	Evaluate real‐word longitudinal performance of Shield test in an average risk population at a second round of testing for CRC detection	Observational prospective cohort	3375	cfDNA, Shield by Guardant Health	Blood	Shield test interrogates cfDNA genomic alterations, aberrant methylation status and fragmentomic patterns.
Colorectal	NCT05485077, started 2022 China	Validate the real‐world results of polygene methylation detection in CRC in a large prospective community cohort	Observational prospective cohort	18000	Polygene methylation on ctDNA	Blood	Polygene methylation detection technology using high‐throughput NGS
Pancreas	NCT06334458, started 2023 France, Italy, Romania and Spain	Pursue early diagnosis for pancreatic cancers in high‐risk asymptomatic subject groups, by developing and validating a comprehensive cancer risk prediction algorithm (CRPA) as a clinical support tool to calculate a personalised risk profile	Interventional non‐randomised open label	170	Clinical risk factors and abnormal methylation changes in ctDNA	Blood	Machine learning algorithm using clinical risk factors and epigenetic biomarker changes to predict risk of pancreatic cancer
Pancreas	NCT06108531, started 2023 China	Evaluate the diagnostic ability of exosomal proteins to identify pancreatic cancer through detection of exosomes in the peripheral blood	Observational prospective case control	400	Protein A from circulating exosomes	Blood	Specific technique not disclosed
Pancreas	NCT06283576, started 2024 Sweden	Measure biomarkers in the following cohorts: histologically proven pancreatic cancer, intraductal papillary mucinous neoplasm (IPMN), ordinary branch duct IPMN	Observational prospective cohort	200	ctDNA, extracellular vesicles, proteomics, metabolomics and CTCs	Blood	Specific technique not mentioned
Pancreas	NCT05556603, started 2022 Ruijin Hospital, China	Development, validation and performance evaluation of combined liquid biopsy assay for early detection of pancreatic cancer	Observational prospective case control	7062	cfDNA methylation, ctDNA mutation, protein markers and miRNA markers	Blood	Multi‐omics approach including NGS, proteomics and PCR
Pancreas	NCT06166147, started 2023 China	Performance of cfDNA methylation‐based model for discriminating pancreatic cancer versus non‐cancer	Observational prospective case control	276	Methylated cfDNA	Blood	Bisulphite sequencing
Pancreas	NCT06456281, started 2024 China	Performance of cfDNA multi‐omics model for discriminating pancreatic cancer versus healthy individuals	Observational prospective case control	480	cfDNA multi‐omics	Blood	Low‐pass whole genome sequencing (LP‐WGS) and hybrid capture‐based targeted methylation sequencing (TMS)
Pancreas	NCT06388967, started 2023 United Stated of America, Japan and South Korea	Validate an exosome‐based miRNA signature for non‐invasive and early detection of PDAC	Observational prospective case control	2000	5 cell‐free and 8 exosome‐miRNAs: PANXEON assay	Blood	qRT‐PCR
Pancreas	NCT05625529, started 2022 United States of America	Compare the performance of ExoVerita™ assay in early detection of PDAC to current standard‐of‐care methods of surveillance	Observational prospective cohort	1000	Extracellular vesicle bound protein biomarkers	Blood	ExoVerita™ assay uses alternating current electric (ACE) field to isolate EVs for differentiated multi‐omics applications and includes an optimised machine learning algorithm to identify a panel of EV‐bound protein biomarkers.
Pancreas	NCT04406831, started 2015 United States of America	Determine miRNAs that may distinguish pancreatic patients from unaffected individuals.	Observational prospective cohort	200	miRNA	Blood	PCR
Pancreas	NCT06803771, started 2025 United Kingdom	Evaluate if the novel diagnostic blood test, called Avantect can early detect pancreatic cancer in patients diagnosed with type 2 diabetes within the last 6 months	Interventional randomised parallel assignment trial	15000	cfDNA; Avantect test	Blood	Uses a combination of 5hmC profiling and whole‐genome sequencing
Pancreas	NCT05500027, started 2021 China	Develop a kit combining sCD58, TGFβ1 and CA19‐9, improve the early diagnosis and treatment rate of pancreatic cancer	Observational prospective case control	2000	sCD58 and TGFbeta1	Blood	ELISA
Pancreas	NCT05847855, started 2023 China	Evaluate the performance of an integrated model using fragmentomic profiles of plasma cfNDA for early detection of pancreatic neuroendocrine tumours	Observational prospective cohort	440	Fragmentomic profiles of cfDNA	Blood	Low‐coverage WGS with machine learning
Biliary	NCT07176962, started 2020 China	Evaluate the application of cfDNA methylation liquid biopsy in the diagnosis and management of biliary tract tumours	Observational prospective case control	1800	Methylated cfDNA	Blood	Specific technique not disclosed
Oesophagus and gastric	NCT06278064, started 2024 China	Identify exosome‐associated protein biomarkers for early‐stage upper GI cancers and validate them using advanced proteomic techniques	Observational retrospective case control	562	Exosome derived protein biomarkers specific to upper GI tumours, detected on exosome membranes	Plasma‐derived exosomes and tissue exosomes (for protein correlation)	Discovery phase: quantitative exosome proteomic analysis (data‐independent acquisition, DIA) and single‐vesicle membrane protein analysis (PBA). Validation phase: quantitative validation using parallel reaction monitoring (PRM) on independent cohorts
Gastric	NCT06726642, started 2024 Canada	To understand the performance of blood based cfDNA analysis aimed at detecting early tumours in patients with hereditary cancer syndromes	Observational prospective case control	1000	cfDNA	Blood	Analysis of cfDNA will involve targeted sequencing of key cancer‐related genes, cell‐free methylated DNA immunoprecipitation and high‐throughput sequencing (cfMeDIP‐seq) and shallow whole genome sequencing (sWGS).
Gastric	NCT06232395, started 2024 China	Develop and validate a non‐invasive detection method for early detection and postoperative monitoring of gastric cancer	Prospective cohort	1197	ctDNA	Blood	Specific technique not disclosed
Gastric	NCT05991947, started 2021 China	To develop key technologies for the early screening of gastric cancer	Prospective cohort	1100	ctDNA, protein biomarkers and cfDNA fragments	Blood	NGS, proteomic analysis and fragmentomics
Oesophagus	NCT05688176, started 2016 Belgium	Perform an explorative search of the transcriptome to detect new circulating diagnostic sensitive and specific biomarkers in patients with BE or	Observational prospective cohort	320	miRNA	Blood	Plan to develop a clinical‐grade qPCR test to validate the identified miRNA biomarkers
Oesophagus and gastric	NCT06346054, started 2024 Belgium	Learn if oncometabolic biomarkers, detected in exhaled breath and blood can identify early‐stage gastro‐oesophageal cancer in patient at risk for gastro‐oesophageal cancer	Non‐randomised interventional parallel assignment	1000	Proteins, ctDNA and volatile organic compounds	Breath and blood	Mass spectrometry and NGS
Liver	NCT04965259, started 2021 Singapore	Validate a panel of circulating miRNA biomarkers to develop an in‐vitro diagnostic (IVD) kit for the detection of early HCC	Observational prospective cohort	2000	Changes in circulating miRNA, gut microbiota and urine metabolome	Blood, urine and stool	miRNA, microbiome and metabolome profiling
Liver	NCT06778317, started 2025 France	Evaluate the role of circulating epigenetic biomarker mSEPT9 in predicting the risk of HCC in patients with cirrhosis	Observational prospective cohort	400	Circulating mSEPT9 Biomarker Testing	Blood	mSEPT9 test: evaluating SEPT9 gene methylation status using a triplicate assay
Liver	NCT06544083, started 2024 China	Establish an early diagnosis model for the diagnosis of liver cancer based on peripheral blood protein indexes and verify its efficacy	Observational prospective cohort	300	Circulating proteins (undisclosed)	Blood	Proteomics
Liver	NCT05199259, started 2022 United States of America	Develop and validate the Helio multi‐analyte blood test	Observational prospective cohort	1200	Methylated cfDNA and serum protein markers (AFP, AFP‐L3% and DCP)	Blood	Helio multi‐analyte test uses cfDNA methylation patterns of 28 genomic regions via NGS based cfDNA methylation in combination with serum protein markers and patient demographics to generate a qualitative test result.
Multi‐cancer	NCT06391749, started 2024 Vietnam	Evaluate the performance of the SPOT‐MAS test in detecting cancer in symptomatic populations	Observational prospective cohort	1000	ctDNA signatures: methylation patterns, fragment length variations, DNA copy number aberrations and end motifs	Blood	Uses a combined approach of targeted and genome‐wide bisulphite sequencing to enable analysis of multiple ctDNA signatures; uses machine learning algorithms for score of ctDNA signal detection
Multi‐cancer	NCT06822413, started 2022 China	Explore if Raman‐based, deep learning‐assisted approach can be used to develop an effective method for early pan‐cancer screening	Observational prospective cohort	1132	Alterations in biochemical composition	Blood	Using deep learning AI algorithms for classifying patients based on Raman spectroscopy analysis of blood samples
Multi‐cancer	NCT06440018, started 2024 China	Develop and implement a blinded validation of a machine learning‐powered, multi‐cancer early detection model	Observational prospective case–control	5350	cfDNA methylation patterns and fragmentomics	Blood	Specific technique not disclosed
Multi‐cancer	NCT06717295, started 2025 Argentina, Nigeria and United Kingdom	Develop and validate an art‐based blood test for early cancer detection and to monitor treatment effectiveness in cancer patients	Observational prospective cohort	6000	Platelet‐derived RNA biomarkers	Blood	Using an AI‐based earl cancer detection tool that profiles RNA biomarkers from platelets and immune cells
Multi‐cancer	NCT05366881, started 2022 United States of America	Train and validate a genome‐wide methylome enrichment platform to detect multiple cancer types and to differentiate amongst cancer types	Observational prospective case–control	7000	Methylated cfDNA	Blood	Epigenomic‐based genome‐wide methylome enrichment platform profiling cfDNA methylation via bisulphite‐free enrichment of methylated CpGs and high‐throughput sequencing
Multi‐cancer	NCT07035587, started 2024 South Korea	Validate a blood‐based method for the early diagnosis and post‐treatment monitoring of multiple cancers	Observational retrospective case control	1200	cfDNA, RNA and protein profiles	Blood	Specific technique not disclosed
Multi‐cancer	NCT06036563, started 2023 China	Screen and differentiate common cancers in participants with or without suspicious lesions	Observational prospective cohort	3200	cfDNA	Blood	Specific technique not disclosed
Multi‐cancer	NCT06790355, started 2025 China	Evaluate the feasibility of blood‐based MCED test in asymptomatic screening cohort	Observational prospective cohort	2527	ctDNA	Blood	Specific technique not disclosed
Multi‐cancer	NCT05155605, started 2021, United States of America and Canada	Evaluate the safety and performance of the GRAIL MCED test in a population of individuals who are eligible for guideline‐recommended cancer screening	Interventional prospective cohort	35885	Methylated cfDNA	Blood	Targeted methylation‐based NGS combined with machine learning algorithms

One primary approach centres on ctDNA methylation analysis, where epigenetic patterns indicative of malignancy are identified through advanced sequencing and machine learning. This helps early detection by pinpointing characteristic chemical modifications on tumour DNA shed into the bloodstream. For instance, in CRC, trials like NCT06258434 (China, CRC‐Appareo) analyse cfDNA methylation, fragment patterns and copy number variations using bisulphite sequencing, aiming to detect precancerous lesions and early‐stage cancer with high specificity. Similarly, pancreatic cancer research, exemplified by NCT06334458 (multi‐country), utilises abnormal ctDNA methylation within machine learning algorithms to predict cancer risk in high‐risk individuals, enabling earlier intervention. Beyond methylation, ctDNA genomic alterations and fragmentomics are crucial, helping early detection by revealing specific mutations, structural changes or abnormal DNA fragmentation patterns from tumours; NCT06059963 (US, Signal‐C™) evaluates a test for CRC combining methylation and fragmentation analysis, seeking to identify early‐stage disease and advanced precancerous lesions.

A second major strategy involves circulating miRNAs, which helps early detection by identifying specific miRNA profiles released by cancer cells into the bloodstream. For CRC, studies such as NCT06342440 (US, DENEB) and NCT06342401 (US, ENCODE) are developing diagnostic miRNA panels via Small RNA‐Seq and qPCR, specifically to detect early‐onset CRC and adenomas. In pancreatic cancer, NCT06388967 (multi‐country, PANXEON assay) validates exosome‐based miRNA signatures using qRT‐PCR to enable minimally invasive early detection. Circulating proteins and peptides represent a third key biomarker class, aiding early detection by identifying abnormal protein levels or forms shed by tumours or produced in response to cancer. For CRC, NCT06955767 (France) investigates MMP‐14 exosomal protein via ELISA to determine a cut‐off value for screening, while NCT07035691 (UK) uses ELISA for circulating progastrin (hPG80) to predict CRC or polyps. Pancreatic cancer studies like NCT05500027 (China) develop kits combining sCD58, TGFβ1 and CA19‐9 to improve early diagnosis rates.

Increasingly, multi‐omics approaches are integrating these diverse biomarkers (cfDNA, miRNA, proteins) to build more comprehensive and robust diagnostic models. This approach helps early detection by combining multiple layers of molecular information, yielding a more sensitive and specific cancer signal than single biomarkers alone. For CRC, NCT06220617 (China) develops a prediction model using cfDNA, miRNA and other markers with NGS and machine learning. For pancreatic cancer, NCT05556603 (China) uses a multi‐omics approach including cfDNA methylation, ctDNA mutation, protein and miRNA markers to develop a combined assay for early detection. Unique approaches also contribute, such as NCT05648240 (France) for CRC, which quantifies CTCs using the ANGLE Parsortix™ system to detect tumour spread, and NCT06346054 (Belgium) for oesophagus and gastric cancer, which investigates oncometabolic biomarkers in breath and blood using mass spectrometry and NGS to identify early‐stage disease. These concerted efforts across various GI cancers, including liver cancer (e.g., NCT05199259 (US) with the Helio test combining cfDNA methylation and serum proteins for early HCC detection) and biliary tract tumours (e.g., NCT07176962 (China) with cfDNA methylation for diagnosis and management), alongside MCED initiatives (e.g., NCT06391749 (Vietnam) analysing diverse ctDNA signatures and NCT06717295 (multi‐country) profiling platelet‐derived RNA to identify multiple cancers from a single blood draw), collectively signify a paradigm shift towards minimally invasive, comprehensive screening strategies for earlier detection and improved patient outcomes.

More importantly, liquid biopsy approaches may be particularly impactful in hereditary cancer settings, such as Lynch syndrome, where lifelong surveillance is required. Recent studies have demonstrated the feasibility of detecting MSI in plasma using ddPCR, enabling identification of early neoplastic changes before clinical or radiographic manifestation.^142^ Such strategies offer a minimally invasive adjunct to endoscopic surveillance and may allow earlier intervention in genetically high‐risk populations.

## 8 CHALLENGES AND FUTURE DIRECTIONS

While liquid biopsies for GI cancer detection are making rapid strides, there are important challenges to address on the path to routine clinical use. Sensitivity at early stages remains a limiting factor – small tumours have very little DNA or cells, so even the best assays may miss a portion of true early cancers. Technological refinements like analysing larger blood volumes, improving DNA extraction efficiency and further error‐correction in sequencing can help. Serial testing (repeating the test over time) might also improve detection rates for indolent early lesions. Specificity is equally critical: false positives can lead to anxiety and invasive follow‐ups. Most current assays achieve >90–99% specificity, but as sensitivity increases, care must be taken to avoid over‐calling benign signals. One thorny issue is clonal haematopoiesis of indeterminate potential (CHIP) – age‐related mutations in blood cells can show DNA that mimics tumour mutations. This can cause false‐positive ctDNA results. Mitigation strategies include parallel sequencing of white blood cells or focusing on methylation markers unaffected by CHIP.

Another challenge is standardisation. Different studies and tests use varying protocols for sample handling, target selection and bioinformatics, making results hard to compare. Establishing standard operating procedures for plasma collection, DNA processing and assay calibration will be essential before liquid biopsy can be widely adopted. Regulatory approval is also a hurdle – demonstrating that a blood test actually improves clinical outcomes (e.g., prevents cancer deaths) often requires large, lengthy trials. So far, the US FDA has cleared only a couple of GI cancer blood tests (SEPT9 and Shield for CRC) as screening tools. Others are available as laboratory‐developed tests (e.g., HelioLiver) but not yet guideline recommended. Ongoing prospective studies like the NCI's SUMMIT and Vanguard trials for multi‐cancer screening will inform how these tests can be implemented at scale. From a clinical standpoint, integration with existing practice is crucial. Liquid biopsy screens will likely be used to complement, not necessarily replace, standard screening. For example, a blood test for CRC can pull in patients who refuse colonoscopy, but any positive result still needs a confirmatory colonoscopy. Similarly, multi‐cancer tests could guide imaging workups to locate an early tumour signal. Equally important is the development of structured, algorithm‐based follow‐up workflows to guide diagnostic evaluation after a positive liquid biopsy result. Clear triage pathways that incorporate clinical risk factors, repeat testing and targeted imaging are essential to prevent unnecessary invasive procedures, mitigate patient anxiety and ensure efficient use of healthcare resources. Beyond analytical performance, successful implementation of liquid biopsy screening depends on cost‐effectiveness, infrastructure readiness and alignment with existing healthcare workflows. While sequencing costs continue to decline, downstream expenditures related to confirmatory testing, imaging and specialist referrals must be considered. Health systems will need to balance screening uptake with diagnostic capacity, establish reimbursement pathways and define risk‐based algorithms to ensure that early detection benefits translate into sustainable population‐level impact.

Looking ahead, the field is moving rapidly towards precision early detection. This involves tailoring screening strategies to individuals based on risk factors (genetic predisposition, cirrhosis, etc.) and using the most appropriate liquid biopsy markers for each context. For high‐risk groups (e.g., hereditary CRC syndromes, chronic pancreatitis patients), intensive liquid biopsy surveillance might catch cancers that current methods miss. Also, continuous monitoring via blood draws could detect recurrence or second primary tumours early in survivors of GI cancers, an extension of the MRD concept to screening. The convergence of liquid biopsy with other modalities – such as combining blood tests with advanced imaging or AI risk prediction models – may yield integrated screening paradigms that drastically improve early detection rates.

## AUTHOR CONTRIBUTIONS

*Conceptualization*: AHZ, ND and PLW; *Literature Review*: MA, CS, HYP, EEG, AG, AFK and ND; *Writing‐oiriginal draft*: MA, CS and ND; *Writing‐review and editing*: MA, CS, ND, AO, DLB, AjG, PLW, AHZ; *Visualization*: CS and ND; *Supervision*: AHZ. All authors read and approved the final manuscript.

## CONFLICT OF INTEREST STATEMENT

Muhammad Anees is severing in a consultant/advisory role for Delfi Diagnostics. Ali H. Zaidi is serving in a consultant/advisory role for Previse, Prognomiq, Gilead, Delfi Diagnostics and BilliontoOne. He has received significant research funding from Eli Lilly, Prognomiq, BilliontoOne, Genece Health, Roche, Myriad Genetics, Delfi Diagnostics, Caris, Natera and Tempus. Ali H. Zaidi has equity interest in Iovance, Previse, Tg Therapeutics, Biocryst, Prognomiq and Gritstone Bio.

## Supporting information



Supporting Information
