# Supplementary material for: Liquid biopsy biomarkers for early detection of gastrointestinal cancers: Current landscape and emerging technologies
Source: Clin Transl Med. 2026 Mar 22;16(3):e70594. doi: 10.1002/ctm2.70594 (PMC13093818; doi:10.1002/ctm2.70594)
Supplement: Supplementary file 1 — Supporting Information [file CTM2-16-e70594-s001.docx]

**Supplementary Table 1:** Common ctDNA mutations associated with GI cancers

| **Genetic mutation** | **Function** | **Pathway** | **Clinical Significance/Targetability** | **Ref.** |
| --- | --- | --- | --- | --- |
| **Oncogenes (Gain-of-function) These genes, when mutated, can promote cell growth and division. A single mutated copy can be sufficient to drive cancer.** | | | | |
| AKT1 | Involved in cell survival and proliferation. | PI3K/AKT/mTOR | Most common actionable AKT1 mutation is E17K, This mutation is associated with mucinous histology and concurrent BRAF V600E mutations in colorectal carcinoma, and it confers primary resistance to anti-EGFR therapy (e.g., cetuximab) | [1, 2] |
| BRAF | A key component of the MAPK signaling pathway, involved in cell growth. | RAS/MAPK | Occurs in approximately 5–10% of metastatic colorectal cancers (CRC), 1–5% of biliary tract cancers, and at lower frequencies in small bowel, pancreatic, and gastric cancers. BRAF V600E is a validated therapeutic target. In metastatic CRC, the combination of BRAF inhibitor (encorafenib) and EGFR inhibitor (cetuximab) .For non-colorectal GI cancers, the combination of dabrafenib (BRAF inhibitor) and trametinib (MEK inhibitor) use for unresectable or metastatic solid tumors with BRAF V600E mutations after prior therapy, with meaningful response rates in biliary tract, small bowel, ampullary, and gastroesophageal cancers. | [3-5] |
| CTNNB1 | Involved in cell adhesion and can promote cell proliferation. | Wnt signaling | Prevalent in HCC (up to 40%)., C less common than APC mutations in CRC but can substitute for APC loss, leading to constitutive β-catenin activation and tumorigenesis. Tegavivint, a TBL1 inhibitor, is in phase 1/2 trials for HCC with CTNNB1 mutations. Combination strategies and dual targeting (e.g., β-catenin and NHERF1) are under investigation. | [6-9] |
| EGFR | A receptor tyrosine kinase that promotes cell growth and survival. | RAS/MAPK (upstream activator) | EGFR is overexpressed in 65–80% of CRC. Aberrant EGFR signaling also plays a role in the pathogenesis of gastroesophageal, pancreatic, and biliary tract cancers, though its clinical impact and targetability vary by tumor type). Monoclonal antibodies targeting EGFR—cetuximab and panitumumab guideline-recommended for RAS wild-type mCRC, either as monotherapy or in combination with chemotherapy (FOLFOX or FOLFIRI). RAS (KRAS/NRAS) and BRAF mutations confer resistance and must be excluded before initiating anti-EGFR therapy. | [10-12] |
| FGFR2 | A receptor tyrosine kinase involved in cell growth and differentiation. | RAS/MAPK (upstream activator, can feed into MAPK) | In intrahepatic cholangiocarcinoma (iCCA), in approximately 9–15% The NCCN recommends routine testing for FGFR2 fusions/rearrangements in unresectable or metastatic iCCA, as several FGFR inhibitors—futibatinib and pemigatinib as first-line therapy. In gastric and GEJ adenocarcinoma, FGFR2 amplification and FGFR2b protein overexpression in 4–30%,. Bemarituzumab, a monoclonal antibody targeting FGFR2b, has shown clinical benefit in phase II trials when combined with chemotherapy, improving overall survival and response rates in FGFR2b-overexpressing, HER2-negative advanced gastric/GEJ Tumor heterogeneity and isoform switching (e.g., FGFR2-IIIc) may impact response and resistance, | [13-18] |
| GNAS | Involved in G protein-coupled receptor signaling. | G protein signaling | GNAS mutations at codon R201, are found in up to 70% of pancreatic IPMNs, 20–50% of appendiceal mucinous neoplasms, and 2–5% of CRC,. In CRC, GNAS mutations are associated with larger tumor size, lymphatic involvement, mucinous differentiation, and often co-occur with KRAS or BRAF mutations. In appendiceal adenocarcinoma, GNAS mutations are linked to poor response to chemotherapy and worse disease event-free survival in metastatic settings .In pancreatic neoplasms, GNAS mutations cooperate with KRAS to drive tumorigenesis and are associated with the intestinal subtype and colloid invasive pattern, which may have a more indolent course. There are currently no approved therapies that directly target mutant GNAS in GI cancers. |  |
| HRAS, KRAS, NRAS | Members of the RAS family, crucial for cell signaling and proliferation. | RAS/MAPK | Present in 30–40% of CRC and are central to tumorigenesis via persistent activation of MAPK/ERK and PI3K/AKT pathways. They are associated with resistance to anti-EGFR monoclonal antibodies (cetuximab, panitumumab), making RAS genotyping mandatory before considering these agents. KRAS G12C mutations (~3–4% of CRC) are now actionable: sotorasib and adagrasib, in combination with anti-EGFR therapy, are FDA-approved for previously treated KRAS G12C-mutant metastatic CRC. NRAS mutations occur in 3–5% of CRC and, like KRAS, predict resistance to anti-EGFR therapy and are associated with poor survival. No direct NRAS inhibitors are approved; management relies on standard chemotherapy and exclusion from anti-EGFR therapy. HRAS mutations are rare in GI cancers but may occur in a small subset. While tipifarnib (a farnesyltransferase inhibitor) is approved for HRAS-mutant head and neck cancers, its role in GI malignancies is investigational. | [19-21] |
| PIK3CA | Catalytic subunit of PI3K, involved in cell growth and survival. | PI3K/AKT/mTOR | PIK3CA encodes the p110α catalytic subunit of PI3K, and activating mutations (most commonly E542K, E545K, H1047R) are found in 10–20% of CRC and 7–25% of gastric cancers. PI3Kα inhibitors (e.g., alpelisib, inavolisib) are not currently FDA-approved in GI cancers. Clinical trials in colorectal and gastric cancer have shown limited efficacy, likely due to frequent co-mutations (KRAS, BRAF, PTEN loss) and intrinsic resistance. | [22-24] |
| TOP2A | Involved in DNA topology. | Cell cycle regulation/DNA repair | Overexpressed in colorectal, gastric, cholangiocarcinoma, and gastroesophageal cancers. TOP2A is the molecular target of anthracyclines (doxorubicin, epirubicin), etoposide, and other topoisomerase II inhibitors, which are used in various GI cancer regimens. | [25-33] |
| **Tumor Suppressor Genes (loss-of-function mutations) These genes normally help control cell growth and division. When both copies are mutated or lost, their protective function is gone, leading to uncontrolled growth.** | | | | |
| APC | Involved in the Wnt signaling pathway and crucial for preventing uncontrolled cell proliferation. | Wnt signaling | Present in up to 80% of sporadic CRC and are the initiating event in the adenoma-carcinoma sequence . While direct targeting is not yet available, therapies aimed at the Wnt/β-catenin pathway and downstream effectors are promising and under active investigation . | [34-37] |
| CDKN2A | Encodes two tumor suppressors (p16INK4a and p14ARF) involved in cell cycle regulation. | Cell cycle regulation/DNA repair | Frequent in pancreatic ductal adenocarcinoma, small bowel adenocarcinoma, ampullary adenocarcinoma, gastric cancer, CRC, and gastrointestinal stromal tumors (GISTs), and are associated with poor overall survival and aggressive disease features. CDK4/6 inhibitors (palbociclib, abemaciclib) are under investigation in GI cancers with CDKN2A loss, with early-phase trials and preclinical data supporting their use, especially when combined with agents targeting co-alterations (e.g., PI3K/mTOR, MEK) (. In pancreatic cancer, dual CDK4/6 and MEK inhibition has shown efficacy in preclinical models . | [38-42] |
| FBXW7 | A ubiquitin ligase involved in degrading oncoproteins. Targets cell cycle regulators for degradation. | Cell cycle regulation/DNA repair | Mutations or reduced expression of FBXW7 are found in 10–16% of CRCs, as well as in gastric, cholangiocarcinoma, and other GI malignancies. FBXW7 loss is associated with advanced stage, lymph node metastasis, poor overall survival, and chemoresistance, particularly in colorectal and gastric cancers). Recent studies show that FBXW7-deficient cancers are selectively sensitive to CDC7 kinase inhibitors, representing a promising synthetic lethal approach . | [43-46] |
| PPP2R1A | A component of protein phosphatase 2A (PP2A). | Many cellular processes | Overexpressed in gastric cancer, In CRC, PP2A activity is commonly decreased, often due to overexpression of endogenous inhibitors (SET, CIP2A) or downregulation of regulatory subunits. PPP2R1A mutations are found in a subset of GISTs via activation of AKT, ERK, and c-KIT signaling. Therapeutic strategies focus on modulating PP2A activity rather than directly targeting PPP2R1A. Small molecules such as FTY720 and DT-061 can restore PP2A activity. In specific contexts (e.g., radiosensitization in pancreatic cancer), PP2A inhibition via PPP2R1A silencing or small molecules (LB100) can enhance the efficacy of radiation by impairing DNA repair and cell cycle checkpoints | [47-50] |
| PTEN | A phosphatase that counteracts PI3K signaling, thus inhibiting cell growth. | PI3K/AKT/mTOR (negative regulator) | Occurs in 5–30% of colorectal and gastric cancers, , cholangiocarcinoma and other GI malignancies. PTEN-deficient tumors show constitutive activation of PI3K/AKT/mTOR signaling, making this pathway a rational therapeutic target. PI3K, AKT, and mTOR inhibitors are under clinical investigation for PTEN-deficient GI cancers, with some trials specifically recruiting patients with PTEN or PIK3CA alterations. Dual PI3K/mTOR inhibitors and combination regimens may be more effective than single agents, given pathway redundancy and feedback activation. In HER2-positive gastroesophageal cancers, PTEN loss predicts resistance to trastuzumab, but combining trastuzumab with PI3K/mTOR inhibitors may overcome resistance. . | [22, 51-55] |
| TP53 | Involved in cell cycle arrest, apoptosis, and DNA repair. | Cell cycle regulation/DNA repair | Occur in up to 74% of CRCs. No direct targeting of mutant p53 is not yet standard, multiple therapeutic strategies—including reactivation, degradation, and immunomodulation—are in development. | [56-60] |
| RNF43 | An E3 ubiquitin ligase. | Wnt signaling (negative regulator) | RNF43 is a negative regulator of Wnt signaling, and loss-of-function mutations (especially frameshift mutations like G659fs) are common in microsatellite instability-high (MSI-H) and BRAF-mutant colorectal cancers, as well as in pancreatic cancers and serrated pathway neoplasia. RNF43 loss-of-function mutations sensitize tumors to Wnt pathway inhibitors, particularly PORCN inhibitors (e.g., RXC004, LGK974). Preclinical and early clinical data show that RNF43-mutant CRC and pancreatic cancers respond to Wnt ligand inhibition, with ongoing trials evaluating RXC004 alone and in combination with immunotherapy(. In metastatic CRC, RNF43 mutations (in microsatellite-stable, BRAF V600E-mutant tumors) predict improved response rates and survival with anti-BRAF/EGFR combinations (e.g., encorafenib plus cetuximab), suggesting RNF43 as a biomarker for patient selection. Specific RNF43 mutations (e.g., G659fs) may confer sensitivity to PI3K/mTOR inhibitors, while RNF43 inactivation in pancreatic cancer enhances BRAF/MEK signaling and increases sensitivity to MEK inhibitors; combined Wnt and MEK inhibition shows synergistic effects. RNF43 mutations are enriched in MSI-H tumors, which are more responsive to immune checkpoint inhibitors. RNF43 status may help stratify patients for immunotherapy and combination approaches. | [61-72] |

References

1. Bleeker, F., et al., *AKT1E17K in human solid tumours.* Oncogene, 2008. **27**(42): p. 5648–5650.

2. Hechtman, J.F., et al., *AKT1 E17K in colorectal carcinoma is associated with BRAF V600E but not MSI-H status: a clinicopathologic comparison to PIK3CA helical and kinase domain mutants.* Molecular Cancer Research, 2015. **13**(6): p. 1003–1008.

3. Driscoll, D., et al., *Prevalence and genomic landscape of BRAF alterations across gastrointestinal cancers*. 2023, American Society of Clinical Oncology.

4. Mahipal, A., et al., *Frequency and outcomes of BRAF alterations identified by liquid biopsy in metastatic, non-colorectal gastrointestinal cancers.* The Oncologist, 2025. **30**(3): p. oyaf044.

5. Kopetz, S., et al., *Encorafenib, binimetinib, and cetuximab in BRAF V600E–mutated colorectal cancer.* New England Journal of Medicine, 2019. **381**(17): p. 1632–1643.

6. Idrissi, Y.A., et al., *Exploring the impact of the β-catenin mutations in hepatocellular carcinoma: an in-depth review.* Cancer Control, 2024. **31**: p. 10732748241293680.

7. Sparks, A.B., et al., *Mutational analysis of the APC/β-catenin/Tcf pathway in colorectal cancer.* Cancer research, 1998. **58**(6): p. 1130–1134.

8. Saponaro, C., et al., *β-catenin knockdown promotes NHERF1-mediated survival of colorectal cancer cells: implications for a double-targeted therapy.* Oncogene, 2018. **37**(24): p. 3301–3316.

9. Li, D., et al., *A phase 1/2 study of the TBL1 inhibitor, tegavivint (BC2059), in patients (pts) with advanced hepatocellular carcinoma (aHCC) with β-catenin activating mutations*. 2024, American Society of Clinical Oncology.

10. Napolitano, S., et al., *Targeting the EGFR signalling pathway in metastatic colorectal cancer.* The Lancet Gastroenterology & Hepatology, 2024. **9**(7): p. 664–676.

11. Quasir Mahmood, M., et al., *The role of epidermal growth factor receptor in the management of gastrointestinal carcinomas: present status and future perspectives.* Current Pharmaceutical Design, 2017. **23**(16): p. 2314–2320.

12. Sepulveda, A.R., et al., *Molecular biomarkers for the evaluation of colorectal cancer: guideline from the American Society for Clinical Pathology, College of American Pathologists, Association for Molecular Pathology, and American Society of Clinical Oncology.* American journal of clinical pathology, 2017. **147**(3): p. 221–260.

13. Valle, J.W., et al., *Biliary tract cancer.* The Lancet, 2021. **397**(10272): p. 428–444.

14. Wainberg, Z.A., et al., *Bemarituzumab in patients with FGFR2b-selected gastric or gastro-oesophageal junction adenocarcinoma (FIGHT): a randomised, double-blind, placebo-controlled, phase 2 study.* The lancet oncology, 2022. **23**(11): p. 1430–1440.

15. Smyth, E.C., et al., *FGFR2b protein overexpression: An emerging biomarker in gastric and gastroesophageal junction adenocarcinoma.* Cancer Treatment Reviews, 2025: p. 102971.

16. Gordon, A., et al., *Targeting FGFR2 positive gastroesophageal cancer: current and clinical developments.* OncoTargets and Therapy, 2022. **15**: p. 1183.

17. Albin, J., et al., *FGFR2-amplified tumor clones are markedly heterogeneously distributed in carcinomas of the upper gastrointestinal tract.* Journal of Cancer Research and Clinical Oncology, 2023. **149**(8): p. 5289–5300.

18. Hashimoto, T., et al., *FGFR2-IIIc isoform detection reveals prognostic prevalence and a functional link to mesenchymal transition in gastric and gastroesophageal junction cancer.* ESMO open, 2025. **10**(11): p. 105851.

19. Takeda, M., et al., *The role of KRAS mutations in colorectal cancer: Biological insights, clinical implications, and future therapeutic perspectives.* Cancers, 2025. **17**(3): p. 428.

20. Caughey, B.A. and J.H. Strickler, *Targeting KRAS-mutated gastrointestinal malignancies with small-molecule inhibitors: A new generation of breakthrough therapies.* Drugs, 2024. **84**(1): p. 27–44.

21. Schirripa, M., et al., *Role of NRAS mutations as prognostic and predictive markers in metastatic colorectal cancer.* International journal of cancer, 2015. **136**(1): p. 83–90.

22. Chong, M.L., et al., *Phosphatidylinositol‐3‐kinase pathway aberrations in gastric and colorectal cancer: Meta‐analysis, co‐occurrence and ethnic variation.* International journal of cancer, 2014. **134**(5): p. 1232–1238.

23. Wang, H., et al., *The role of PIK3CA gene mutations in colorectal cancer and the selection of treatment strategies. Front Pharmacol. 2024; 15: 1494802*.

24. Fernandes, M.S., J.M. Sanches, and R. Seruca, *Targeting the PI3K signalling as a therapeutic strategy in colorectal cancer.* Targeted Therapy of Colorectal Cancer Subtypes, 2019: p. 35–53.

25. Wang, X., et al., *Oncogenic role and potential regulatory mechanism of topoisomerase IIα in a pan-cancer analysis.* Scientific Reports, 2022. **12**(1): p. 11161.

26. Zhao, F., et al., *Oncogenetic function and prognostic value of DNA topoisomerase II alpha in human malignances: a pan-cancer analysis.* Frontiers in Genetics, 2022. **13**: p. 856692.

27. Coss, A., et al., *Increased topoisomerase IIα expression in colorectal cancer is associated with advanced disease and chemotherapeutic resistance via inhibition of apoptosis.* Cancer letters, 2009. **276**(2): p. 228–238.

28. Ong, K.H., et al., *Prognostic significance of DNA topoisomerase II Alpha (TOP2A) in cholangiocarcinoma.* Frontiers in Bioscience-Landmark, 2023. **28**(4).

29. Fogt, F., et al., *Topoisomerase II alpha expression in normal, inflammatory, and neoplastic conditions of the gastric and colonic mucosa.* Modern pathology: an official journal of the United States and Canadian Academy of Pathology, Inc, 1997. **10**(4): p. 296–302.

30. Zhou, T., Y. Niu, and Y. Li, *Advances in research on malignant tumors and targeted agents for TOP2A.* Molecular Medicine Reports, 2024. **31**(2): p. 50.

31. Ali, Y. and S. Abd Hamid, *Human topoisomerase II alpha as a prognostic biomarker in cancer chemotherapy.* Tumor Biology, 2016. **37**(1): p. 47–55.

32. Kanta, S.Y., et al., *Topoisomerase IIα gene amplification in gastric carcinomas: correlation with the HER2 gene. An immunohistochemical, immunoblotting, and multicolor fluorescence in situ hybridization study.* Human pathology, 2006. **37**(10): p. 1333–1343.

33. Nygård, S.B., et al., *Underpinning the repurposing of anthracyclines towards colorectal cancer: assessment of topoisomerase II alpha gene copy number alterations in colorectal cancer.* Scandinavian Journal of Gastroenterology, 2013. **48**(12): p. 1436–1443.

34. Aghabozorgi, A.S., et al., *Role of adenomatous polyposis coli (APC) gene mutations in the pathogenesis of colorectal cancer; current status and perspectives.* Biochimie, 2019. **157**: p. 64–71.

35. Zhang, L., et al., *Selective targeting of mutant adenomatous polyposis coli (APC) in colorectal cancer.* Science translational medicine, 2016. **8**(361): p. 361ra140–361ra140.

36. Samadder, N.J., et al., *Effect of sulindac and erlotinib vs placebo on duodenal neoplasia in familial adenomatous polyposis: a randomized clinical trial.* Jama, 2016. **315**(12): p. 1266–1275.

37. Gerner, E.W., et al., *A comprehensive strategy to combat colon cancer targeting the adenomatous polyposis coli tumor suppressor gene.* Annals of the New York Academy of Sciences, 2005. **1059**(1): p. 97–105.

38. Kimura, H., et al., *The role of inherited pathogenic CDKN2A variants in susceptibility to pancreatic cancer.* Pancreas, 2021. **50**(8): p. 1123–1130.

39. Delgado-de la Mora, J., et al., *Novel structural variants that impact cell cycle genes are elucidated in metastatic gastrointestinal stromal tumors.* Pathology-Research and Practice, 2025. **266**: p. 155782.

40. Mikhail, S., C. Albanese, and M.J. Pishvaian, *Cyclin-dependent kinase inhibitors and the treatment of gastrointestinal cancers.* The American journal of pathology, 2015. **185**(5): p. 1185–1197.

41. Kato, S., et al., *Targeting G1/S phase cell-cycle genomic alterations and accompanying co-alterations with individualized CDK4/6 inhibitor–based regimens.* JCI insight, 2021. **6**(1): p. e142547.

42. Christenson, E.S., E. Jaffee, and N.S. Azad, *Current and emerging therapies for patients with advanced pancreatic ductal adenocarcinoma: a bright future.* The Lancet Oncology, 2020. **21**(3): p. e135–e145.

43. Afolabi, H.A., et al., *Targeted variant prevalence of FBXW7 gene mutation in colorectal carcinoma propagation. The first systematic review and meta-analysis.* Heliyon, 2024. **10**(11).

44. Sun, J., Y.-K. Bai, and Z.-G. Fan, *Role of FBXW7 expression in gastric cancer: Meta‑analysis and bioinformatics analysis.* Oncology Letters, 2023. **25**(5): p. 184.

45. Wang, J., et al., *Loss of Fbxw7 synergizes with activated Akt signaling to promote c-Myc dependent cholangiocarcinogenesis.* Journal of hepatology, 2019. **71**(4): p. 742–752.

46. Baxter, J.S., et al., *Cancer‐associated FBXW7 loss is synthetic lethal with pharmacological targeting of CDC7.* Molecular Oncology, 2024. **18**(2): p. 369–385.

47. Cristóbal, I., et al., *PP2A inhibition is a common event in colorectal cancer and its restoration using FTY720 shows promising therapeutic potential.* Molecular cancer therapeutics, 2014. **13**(4): p. 938–947.

48. Cai, Z., et al., *Protein phosphatase 2a inhibits gastric Cancer cell glycolysis by reducing MYC signaling.* Cell Biochemistry and Biophysics, 2023. **81**(1): p. 59–68.

49. Toda-Ishii, M., et al., *Clinicopathological effects of protein phosphatase 2, regulatory subunit A, alpha mutations in gastrointestinal stromal tumors.* Modern Pathology, 2016. **29**(11): p. 1424–1432.

50. Wei, D., et al., *Inhibition of protein phosphatase 2A radiosensitizes pancreatic cancers by modulating CDC25C/CDK1 and homologous recombination repair.* Clinical Cancer Research, 2013. **19**(16): p. 4422–4432.

51. Salvatore, L., et al., *PTEN in colorectal cancer: shedding light on its role as predictor and target.* Cancers, 2019. **11**(11): p. 1765.

52. Serebriiskii, I.G., et al., *Comprehensive characterization of PTEN mutational profile in a series of 34,129 colorectal cancers.* Nature communications, 2022. **13**(1): p. 1618.

53. De Roock, W., et al., *KRAS, BRAF, PIK3CA, and PTEN mutations: implications for targeted therapies in metastatic colorectal cancer.* The lancet oncology, 2011. **12**(6): p. 594–603.

54. Lim, H.J., P. Crowe, and J.-L. Yang, *Current clinical regulation of PI3K/PTEN/Akt/mTOR signalling in treatment of human cancer.* Journal of cancer research and clinical oncology, 2015. **141**(4): p. 671–689.

55. Yokoyama, D., et al., *PTEN is a predictive biomarker of trastuzumab resistance and prognostic factor in HER2-overexpressing gastroesophageal adenocarcinoma.* Scientific Reports, 2021. **11**(1): p. 9013.

56. Yan, S., et al., *p53 in colorectal cancer: from a master player to a privileged therapy target.* Journal of Translational Medicine, 2025. **23**(1): p. 684.

57. Liebl, M.C. and T.G. Hofmann, *The role of p53 signaling in colorectal cancer.* Cancers, 2021. **13**(9): p. 2125.

58. Fesler, A., N. Zhang, and J. Ju, *The expanding regulatory universe of p53 in gastrointestinal cancer.* F1000Research, 2016. **5**: p. 756.

59. Kim, K.M., et al., *Clinical significance of p53 protein expression and TP53 variation status in colorectal cancer.* BMC cancer, 2022. **22**(1): p. 940.

60. Wang, H., et al., *Targeting p53 pathways: Mechanisms, structures and advances in therapy.* Signal transduction and targeted therapy, 2023. **8**(1): p. 92.

61. Yan, H.H., et al., *RNF43 germline and somatic mutation in serrated neoplasia pathway and its association with BRAF mutation.* Gut, 2017. **66**(9): p. 1645–1656.

62. Bond, C.E., et al., *RNF43 and ZNRF3 are commonly altered in serrated pathway colorectal tumorigenesis.* Oncotarget, 2016. **7**(43): p. 70589.

63. Vogel, A., et al., *Association of RNF43 Genetic Alterations With BRAFV600E and MSIhigh in Colorectal Cancer.* JCO Precision Oncology, 2024. **8**: p. e2300411.

64. Yamamoto, D., et al., *Characterization of RNF43 frameshift mutations that drive Wnt ligand‐and R‐spondin‐dependent colon cancer.* The Journal of Pathology, 2022. **257**(1): p. 39–52.

65. Yu, J., et al., *The functional landscape of patient-derived RNF43 mutations predicts sensitivity to Wnt inhibition.* Cancer research, 2020. **80**(24): p. 5619–5632.

66. Jiang, X., et al., *Inactivating mutations of RNF43 confer Wnt dependency in pancreatic ductal adenocarcinoma.* Proceedings of the National Academy of Sciences, 2013. **110**(31): p. 12649–12654.

67. Elez, E., et al., *RNF43 mutations predict response to anti-BRAF/EGFR combinatory therapies in BRAF V600E metastatic colorectal cancer.* Nature medicine, 2022. **28**(10): p. 2162–2170.

68. Moretto, R., et al., *Predictive impact of RNF43 mutations in patients with proficient mismatch repair/microsatellite stable BRAFV600E-mutated metastatic colorectal cancer treated with target therapy or chemotherapy.* JCO Precision Oncology, 2023. **7**: p. e2300255.

69. Fang, L., et al., *RNF43 G659fs is an oncogenic colorectal cancer mutation and sensitizes tumor cells to PI3K/mTOR inhibition.* Nature communications, 2022. **13**(1): p. 3181.

70. Hsu, S.H., et al., *RNF43 inactivation enhances the B‐RAF/MEK signaling and creates a combinatory therapeutic target in cancer cells.* Advanced Science, 2024. **11**(12): p. 2304820.

71. Huo, X., et al., *RNF43 in cancer: Molecular understanding and clinical significance in immunotherapy.* The Journal of Gene Medicine, 2024. **26**(8): p. e3729.

72. Xu, Y., et al., *Pan-cancer analysis identifies RNF43 as a prognostic, therapeutic and immunological biomarker.* European Journal of Medical Research, 2023. **28**(1): p. 438.
